# Virome diversity shaped by genetic evolution and ecological landscape of *Haemaphysalis longicornis*

**DOI:** 10.1186/s40168-024-01753-9

**Published:** 2024-02-21

**Authors:** Run-Ze Ye, Yu-Yu Li, Da-Li Xu, Bai-Hui Wang, Xiao-Yang Wang, Ming-Zhu Zhang, Ning Wang, Wan-Ying Gao, Cheng Li, Xiao-Yu Han, Li-Feng Du, Luo-Yuan Xia, Ke Song, Qing Xu, Jing Liu, Nuo Cheng, Ze-Hui Li, Yi-Di Du, Hui-Jun Yu, Xiao-Yu Shi, Jia-Fu Jiang, Yi Sun, Tick Genome and Microbiome Consortium (TIGMIC), Xiao-Ming Cui, Shu-Jun Ding, Lin Zhao, Wu-Chun Cao

**Affiliations:** 1 Institute of EcoHealth, School of Public Health, Cheeloo College of Medicine, Shandong University, Jinan, Shandong People’s Republic of China; 2 State Key Laboratory of Pathogen and Biosecurity, Beijing Institute of Microbiology and Epidemiology, Beijing, People’s Republic of China; 3 Department of Epidemiology, School of Public Health, Cheeloo College of Medicine, Shandong University, Jinan, People’s Republic of China; 4 Shandong Provincial Key Laboratory of Communicable Disease Control and Prevention, Department of Communicable Disease Control and Prevention, Shandong Center for Disease Control and Prevention, Jinan, Shandong People’s Republic of China; 5 Research Unit of Discovery and Tracing of Natural Focus Diseases, Chinese Academy of Medical Sciences, Beijing, People’s Republic of China

## Abstract

**Background:**

*Haemaphysalis longicornis* is drawing attentions for its geographic invasion, extending population, and emerging disease threat. However, there are still substantial gaps in our knowledge of viral composition in relation to genetic diversity of *H. longicornis* and ecological factors, which are important for us to understand interactions between virus and vector, as well as between vector and ecological elements.

**Results:**

We conducted the meta-transcriptomic sequencing of 136 pools of *H. longicornis* and identified 508 RNA viruses of 48 viral species, 22 of which have never been reported. Phylogenetic analysis of mitochondrion sequences divided the ticks into two genetic clades, each of which was geographically clustered and significantly associated with ecological factors, including altitude, precipitation, and normalized difference vegetation index. The two clades showed significant difference in virome diversity and shared about one fifth number of viral species that might have evolved to “generalists.” Notably, *Bandavirus dabieense*, the pathogen of severe fever with thrombocytopenia syndrome was only detected in ticks of clade 1, and half number of clade 2-specific viruses were aquatic-animal-associated.

**Conclusions:**

These findings highlight that the virome diversity is shaped by internal genetic evolution and external ecological landscape of *H. longicornis* and provide the new foundation for promoting the studies on virus-vector-ecology interaction and eventually for evaluating the risk of *H. longicornis* for transmitting the viruses to humans and animals.

Video Abstract

**Supplementary Information:**

The online version contains supplementary material available at 10.1186/s40168-024-01753-9.

## Introduction

*Haemaphysalis longicornis*, a longhorned tick native in eastern Asia, has invaded and spread to new regions worldwide, including the USA [1–3]. Partly because of its genomic characteristics and behavioral preferences, the tick species might affect more extensive regions, where it has never been recorded before [4]. In recent years, *H. longicornis* is drawing increasing attention for its geographic invasion, extending population, and emerging disease threat [1, 5]. *H. longicornis* can harbor a wide spectrum of pathogens that cause diseases in humans, wildlife, and livestock. At least 30 human pathogens are known to be associated with the tick globally, involving *Rickettsia*, *Anaplasma*, *Ehrlichia*, *Borrelia*, *Babesia*, *Francisella*, *Coxiella*, and viruses [5]. Particularly, several pathogens have been recently detected among the invasive tick species in the USA [6–10], and more and more *H. longicornis*-associated viruses have been continuously recognized around the world [11–15], indicating the potential risk for transmission of emerging viruses by the tick species.

However, there are still substantial gaps in our knowledge of viral composition in relation to genetic diversity of *H. longicornis* and ecological factors, which are important for us to understand interactions between virus and vector, as well as between vector and ecological elements. In this study, we conducted a meta-transcriptomic investigation of *H. longicornis* in Shandong Province of China, where has an area of 158,000 square kilometers with over 100 million population, and the predominant tick species is *H. longicornis* [5]*.* The objectives of this study were to characterize the virome of *H. longicornis*, to explore the population structure and genetic diversity of the tick species in the region, to investigate the relationship between viromic diversity and genetic evolution of tick population, to identify the ecological factors influencing the genetic diversity of *H. longicornis*, and ultimately to better understand the potential threat of *H. longicornis* to public and veterinary health.

## Methods

### Sample collection

*H. longicornis* were collected across Shandong Province, China from June 2018 to July 2022. The latitude and longitude of each collection site were recorded at the time of tick collection. Ticks were collected by dragging a standard 1 m^2^ flannel flag over vegetation or from domestic animals such as cows, sheep, goats, and dogs. The species, gender, and developmental stage of each tick were identified by an entomologist. Adult *H. longicornis* were included in this study and divided into pools on the basis of sex, sampling sites, and blood-feeding status.

### Library preparation and sequencing

Total DNA and RNA were extracted from pools of *H. longicornis* using the AllPrep DNA/RNA mini kit (Qiagen). Briefly, ticks were homogenized in RLT solution under liquid nitrogen. The homogenate was then incubated at 55 °C for 10 min with proteinase K (Qiagen) and centrifuged for 30 s at 15,000 g. The homogenized lysate was transferred to an AllPrep DNA spin column and centrifuged for 30 s at 8000 g. The flow-through was used for RNA purification following the manufacturer’s instructions. The AllPrep DNA spin column was set aside for later DNA purification used for other study.

The purified RNA was quantified using Qubit 4.0 fluorometer, and RNA quality was assessed using an Agilent Bioanalyzer 2200 (Agilent). The ribosomal RNA was removed using RiBo-Zero Gold rRNA removal reagents (Human/Mouse/Rat) (Illumina). Then, the sequencing library was prepared following the Illumina standard protocol. Paired-end (2 × 150 bp) transcriptome sequencing (RNA-seq) of the RNA library was conducted on an Illumina NovaSeq 6000 platform at Novogene Tech.

### Viral contig assembly and annotation

The AfterQC (V2.3.3) [16] was used to remove low-quality and short reads in Illumina reads. The remaining clean reads were compared against *H. longicornis* genome (GCA_013339765 BIME_HaeL_1.3) using Bowtie2 (v2.3.5.1) [17] to remove reads associated with the host genome. Unmapped reads were de novo assembled using Trinity (v2.13.2) [18]. All assembled contigs were compare against the NCBI nonredundant protein database (nr, 2023/3/12 version) using Diamond BLASTx (v2.0.13) [19] and against the NCBI nucleotide sequence database (nt, 2023/3/04 version) using blastn (v2.12.0 +) [20] to identify virus-associated contigs based on the top BLAST hits of each contig. A significant hit was defined by an *E*-value smaller than 1 × 10^−5^.

The reads remaining after *H. longicornis* genome removed were mapped to NCBI nucleotide sequence database by using Kraken2 (v2.1.2) [21], and virus abundance for ach family was quantified as the number of mapped reads per million total reads in the library (RPM). The clustering analysis for all libraries was performed using the ComplexHeatmap [22] package in R. We focused our ecological analysis on alpha diversity. The Shannon index was calculated for each sample using the Rhea alpha diversity script [23] in the R software. Two-tailed Wilcoxon’s rank-sum tests were used to determine statistically significant differences in gender, blooding-feeding status, and genetic clades.

All putative novel viruses belonging to genus were provisionally denominated as “Cheeloo,” due to the study conducted by the researchers at Cheeloo College of Medicine, Shandong University, China, followed by the viral genus name, and other putative novel viruses were provisionally denominated as “Cheeloo” plus virus-related family name excluding “viridae” characters or virus-related order name excluding “virales” characters. Viruses of superclades were named only using “Cheeloo” plus “tick virus.”

Potential open reading frames (ORFs) in the viral sequences were predicted using Geneious prime v2023.0.4 (https://www.geneious.com) and were further compared with reference sequences. The RNA-dependent RNA polymerase (RdRp) domain was identified by comparisons to the Conserved Domain Database (https://www.ncbi.nlm.nih.gov/Structure/cdd/wrpsb.cgi). Novel viral genomes were confirmed by checking reads coverage and continuity using Bowtie2.

### Genetic evolution analysis of *H. longicornis* based on mitochondrial sequences

Assembled contigs of each sample were aligned to *H. longicornis* reference genome (GWHAMMI00002676) using Bowtie2 (v2.3.5.1) and SAMtools (v1.14) [24]. Variants were called by BCFtools (v1.15.1) [25]. Variant sites with quality scores ≥ 20 was kept for subsequent analysis. Based on the called variants, we generated the mitochondrial sequence of each library using vcf2phylip (v2.8) [26] and built maximum likelihood (ML) trees with 1000 bootstrap replicates using IQ-TREE [27] with the PMB + F + ASC + R2 substitution model according to BIC.

### Comparative analysis between genetic clade of *H. longicornis* and ecological factors

The spatial distribution of ticks in different clades was displayed after estimating the inverse distance weighting (IDW) with ArcGIS software (10.7). Data of ecological variables including elevation, annual average pressure, annual average wind speed, annual precipitation, annual average relative humidity, annual evaporation, annual average temperature, annual average ground surface temperature, annual sunshine duration, and normalized difference vegetation index (NDVI) were extracted from the Institute of Geographic Sciences and Natural Resources Research. Spearman test was used to evaluate correlation of variables with each other. A correlation coefficient greater than 0.7 was considered to be a strong correlation between variables. Variance inflation factor (VIF) values were estimated using car package [28] in R to measure the degree of multicollinearity among the variables. A variable with a VIF of > 5 was considered indicative of multicollinearity and excluded. Then, the generalized linear regression model (GLM) of logit link was used to determine ecological factors influencing distribution of clade 1 and 2 ticks. A *P*-values < 0.05 was considered statistically significant.

To estimate the abundance of the corresponding virus species in the library where the sequence was assembled, a read mapping approach was used by Bowtie2. The remaining reads after host genome filtering were mapped to assembled viral contigs. We acquired the read counts of each viral RNA from mapping results and performed within-sample normalization (reads per million/viral reads) to compare samples.

### Phylogenetic analysis

The longest representative sequence for each cluster was aligned with downloaded reference proteins belonging to the same viral family or order using the E-INS-i algorithm with the implementation of MAFFT (v7.490) [29]. Ambiguously aligned regions were trimmed using TrimAl (v1.4) [30], and short contigs that did not align to reference genomes were removed before constructing the appropriate alignments for downstream phylogeny. The IQ-TREE (v2.2.2.3) algorithm [27] was used to determine the best-fit amino acid or nucleotide substitution model based on each multiple sequence alignment, and the ML phylogenetic trees were subsequently constructed based on sequence alignment using the IQ-TREE with 1000 bootstrap replicates. The ggtree [31], phangorn [32], treeio [33], and ggplot2 [34] packages in R were used to visualize the trees and determine the midpoint as the root of the phylogenetic tree.

## Results

### Virome diversity of *H. longicornis*

**Fig. 1:**
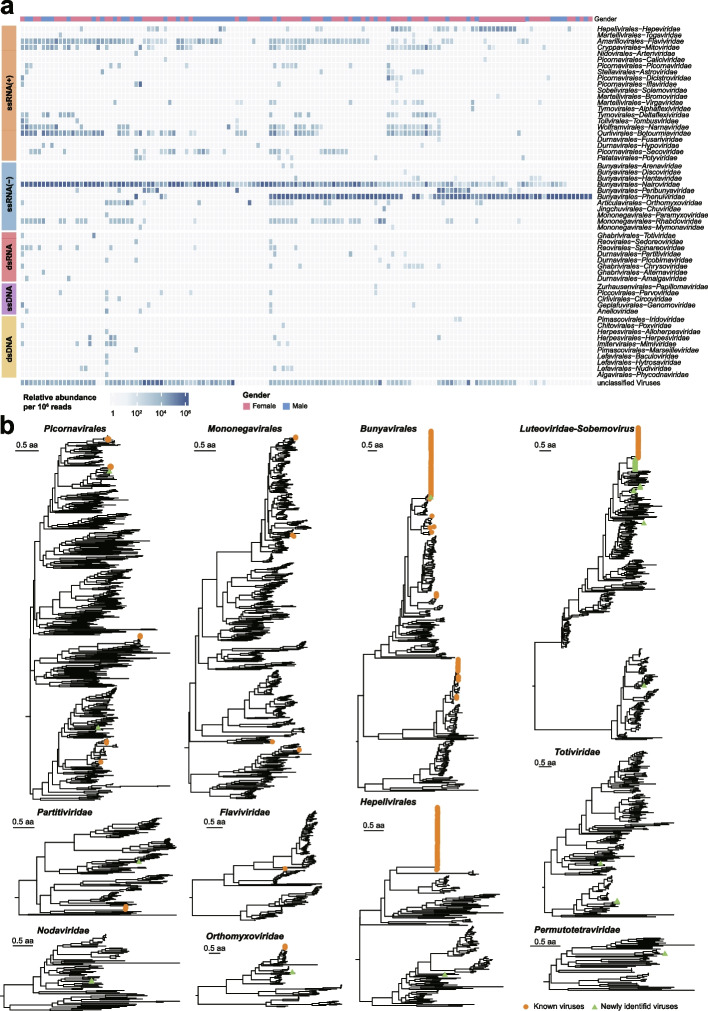
Virome diversity of *H. longicornis.* **a** Virus relative abundance profile across *H. longicornis* libraries. Each cell in the heat map represents the normalized number of reads belonging to the given virus order or family according to Kraken2 annotation. All families were grouped according to Baltimore classification, including ssRNA( +), ssRNA( −), dsRNA, ssDNA, dsDNA, and unclassified viruses. The gender of each sample was indicated in the corresponding colors on the top. **b** Phylogenetic trees were constructed on the basis of RdRp protein for RNA viruses. Newly identified viruses in this study are labeled with green solid triangle; known viruses are labeled with orange solid circles. Families without assembled contigs containing the RdRp domain were not displayed in **b**, although the viral reads were detected in **a**

A meta-transcriptomics survey on virome of *H. longicornis* was conducted in Shandong Province of eastern China. Adult *H. longicornis* samples were collected from 14 prefectures or cities across the province during 2018–2022 and pooled according to sex, collection site, and blood-feeding status for extraction of total RNA. As a result, 136 libraries were successfully constructed for meta-transcriptome sequencing, and a total of 3.6 × 10^9^ 100–150 paired-end reads were obtained. After quality control and removing *H. longicornis* genome reads, 1.49 × 10^7^ viral reads were identified, representing 2.71% of the total clean reads. The percentage of viral reads in each library varied from 0.04 to 43.09% with the interquartile range (IQR) of 0.16–6.50% (Supplementary Table 1). The bacteriophages were then removed for downstream analyses. The remaining viral reads could be divided into 56 established viral families and a group of unclassified viruses (Fig. 1a). The prevalence and abundance greatly varied among viral families. Both positive and negative single-stranded RNA (ssRNA) viruses were more prevalent with relatively greater abundance in comparison double-strand RNA (dsRNA) and DNA viruses. Among the 11 families of ssRNA(-) viruses, families *Nairoviridae* and *Phenuiviridae* in the order *Bunyavirales* were most commonly detected with high abundance and prevalence among the 136 tick samples. In addition, viral reads with unclassified viruses were detected in most tick samples. The virome diversity was not statistically different between male and female ticks and significantly higher in unfed than fed ticks (*P* < 0.0001, Supplementary Fig. 1).

After de novo assembling, a total of 26,611 contigs could be assigned to viral sequences, from which 508 non-redundant RNA virus sequences were assembled for downstream analysis, and deposited into GenBank (Supplementary Table 2). We performed phylogenetic analyses based on the amino acid (aa) of the most conserved RdRp protein (Fig. 1b). We classified the viruses according to the demarcation criteria issued by the International Committee on Taxonomy of Viruses (https://talk.ictvonline.org/ictv-reports/ictv_online_report/). In case the viruses could not be easily categorized according to the scheme of virus classification, we used a threshold of aa identity of 90% for RdRp [35, 36]. As a result, we identified 48 virus species, 26 of which were known viruses belonged to 14 families. The remaining 22 viruses were newly recognized, among which 19 fell into 11 currently established families, while the other three RNA viruses fell outside all known viral families in the phylogenies, which were grouped into putative *Solemoviridae-Tombusviridae* and *Nodaviridae-Solemoviridae* ‘superclades’ with the currently defined virus orders, families, and floating genera by using a previously adopted tactic [35].

### Two genetic clades of *H. longicornis* with distinguishing ecological landscape

**Fig. 2:**
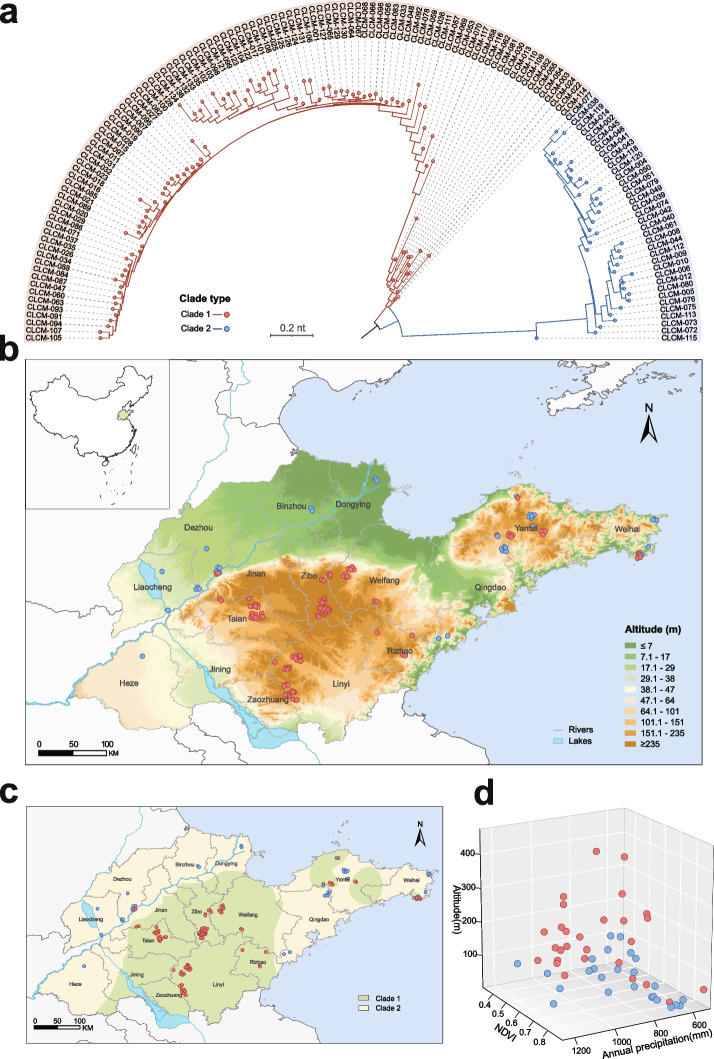
Genetic clade and distribution of *H. longicornis* in relation to ecological factors. **a** Phylogenetic tree of *H. longicornis* based on mitochondrial variants. The red and blue branches represent genetic clade 1 and clade 2, respectively. **b** Spatial distribution of tick collection. The background colors range from green to brown, indicating gradual elevation. The red and blue points represent collection sites of clade 1 and clade 2, respectively. **c** Spatial clustering of each *H. longicornis* clade based on the inverse distance weighting (IDW) analysis. The green-shaded areas are the clusters of ticks in clade 1, and the yellow-shaded areas are the clusters of ticks in clade 2. **d** Three-dimensional scatter plot of tick samples in relation to the three ecological variables. The red and blue points represent clade 1 and clade 2, respectively

The unique ability of *H. longicornis* to rapidly invade new areas and explosively proliferate in its established ranges [5] prompted us to investigate genetic diversity of the tick species. Furthermore, our previous study suggests that different genetic populations of *H. longicornis* exhibit distinctive ecogeographical distribution with various positive rates of bacteria [4]. In this study, we figured out the genetic diversity of *H. longicornis* by phylogenetic analysis on the bases of single-nucleotide polymorphisms of mitochondria from the meta-transcriptome data. We found that the 136 *H. longicornis* samples were clustered into two distinct genetic clades (Fig. 2a), which substantially reflected their different geographical distribution: clade 1 included ticks mainly from the mountain area and clade 2 mainly from Jiaodong Peninsula and northern plain of Shandong Province (Fig. 2b). The inverse distance weighting (IDW) analysis revealed the distinctive geographical distribution of the ticks in each clade (Fig. 2c), suggesting the genetic clades are associated with special ecological landscape.

To determine the ecological factors influencing distribution of ticks in clades 1 and 2, we performed quantitative analyses. After excluding the indicative variables with multicollinearity, multivariable analysis using generalized linear regression model (GLM) of logit link revealed that the distribution of tick clades was significantly associated with altitude, annual precipitation, and NDVI (Fig. 2d). In comparison to the ticks in clade 2, the ticks in clade 1 distributed in higher areas with an adjusted odds ratio (aOR) of 1.027 (95% confidence interval (CI), 1.015–1.043; *P* < 0.001). Clade 1 areas had significantly higher annual precipitation (aOR, 1.005; 95% CI 1.001–1.010; *P* = 0.033) and lower NDVI (aOR, 0.036; 95% CI 0.004–0.409; *P* = 0.036). These findings imply that the above three ecological elements play a role in evolutionary trajectory of each *H. longicornis* clade by shaping their specific habitats [37, 38]. Further investigations of diverse ecosystems of different tick species are needed for generalization and better understanding of this issue.

### Viral diversity associated with genetic clades of *H. longicornis*

**Fig. 3:**
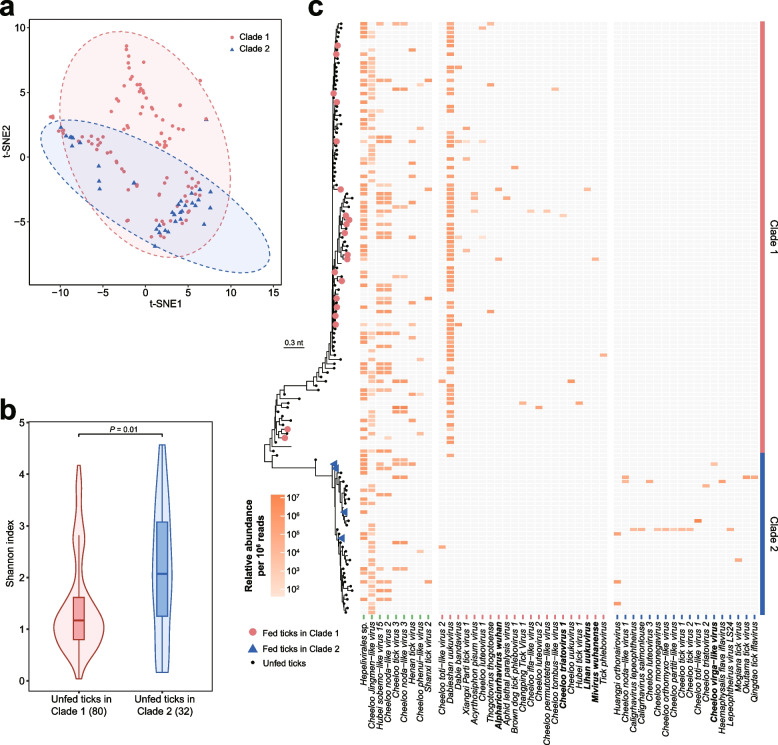
Virus diversity in genetic clades of *H. longicornis*. **a** Between-group clustering of viromes between genetic clades of *H. longicornis* by t-SNE analysis. **b** Shannon indexes of tick viromes between genetic clades of unfed *H. longicornis* (*n*_clade1_ = 80, *n*_clade2_ = 32). Boxplot elements: center line, median; box limits, upper and lower quartiles; whiskers (error bars), the highest and lowest points within 1.5 interquartile range of the upper and lower quartiles. The *P*-value was calculated using a two-sided Wilcoxon rank-sum test. **c** Abundance of each virus identified in this study. The fed tick samples in clade 1 are indicted by red solid circles, and those in clade 2 are indicated by blue solid triangles. The viruses shown in bold is only present in fed tick samples. Each cell in the heat map represents the normalized abundance of viral reads

We compared virome diversity in relation to genetic clades of *H. longicornis* ticks by estimating the operational taxonomic units (OTUs) of viruses in the meta-transcriptome data and found that the virome was distinctive between the two clades of ticks according to t-distributed stochastic neighbor embedding (t-SNE) (Fig. 3a). Considering the virome diversity between fed and unfed ticks was significantly different (*P* < 0.0001) as shown in Supplementary Fig. 1, we excluded the 20 fed ticks from clade 1 and four from clade 2 for the α diversity of virome qualified by Shannon index to avoid possible inhibitory from host blood. As a result, unfed ticks in clade 1 showed significantly lower virus diversity than those in clade 2 (*P* = 0.01) (Fig. 3b).

To further clarify the virus diversity between the two clades, we estimated normalized relative abundance of each virus in each tick sample (Fig. 3c) and subsequently calculated the positive rate with 95% CI of each recognized virus in the 136 tick samples (Supplementary Table 3). Both abundance and prevalence of each identified virus greatly varied among either the tick samples as a whole or ticks in different clades. The 48 virus species recognized in this study could be divided into three categories: viruses (10) presented in both clades, viruses (20) only in clade 1, and viruses (18) only in clade 2. Generally speaking, the 10 viruses shared by the two clades had obviously higher prevalences, often with higher abundance in each sample, while the viruses specific to each clade showed relatively low prevalence except for *Uukuvirus dabieshanense* in clade 1 (Supplementary Table 3). Furthermore, four and one viruses were only detected in fed ticks in clade 1 and clade 2, respectively (Fig. 3c). We could not distinguish whether these viruses originated from the blood of animal hosts based on current results, because 12 and 17 viruses were only found in unfed ticks in clade 1 and clade 2, respectively. These findings suggest that the virus load and prevalence are determined by the genetic factor of ticks, which might be influenced by the ecological habitats they are infesting [39]. The obvious genetic diversities of both ticks and tick-associated viruses indicate that the study on vector-pathogen interactions should be enhanced to better understand tick-borne pathogen evolution and ecology.

### Relatively high prevalence of viruses shared by the two genetic clades of *H. longicornis*

**Fig. 4:**
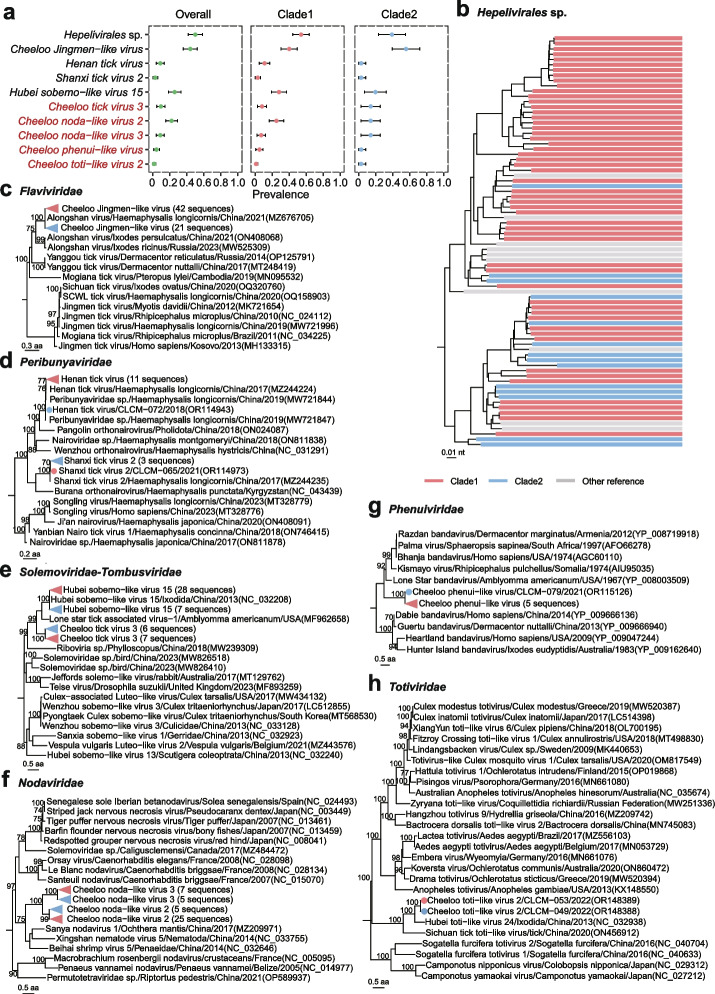
Phylogenetic analysis of viruses in both clades of *H. longicornis*. **a** The prevalence with 95% confidence interval (CI) of each virus. The virus species name shown in red are newly discovered in this study. **b** Phylogeny of viruses in the family *Hepelivirales* sp. based on amino acid sequence of RNA-dependent RNA polymerase (RdRp) gene. Tree s are colored according to the genetic clade of libraries which viral sequences collected from: The red strips indicate tick samples from clade 1, the blue strips represent ticks from clade 2, and the gray strips are reference sequences. **c** Phylogeny of viruses in the group of Jingmenvirus based on amino acid sequence of VP1b protein. **d** Phylogeny of viruses in the family *Peribunyaviridae* based on amino acid sequence of RdRp protein. **e** Phylogeny of viruses between the families *Solemoviridae* and *Tombusviridae* based on amino acid sequence of RdRp protein. **f** Phylogeny of viruses in the family *Nodaviridae* based on amino acid sequence of capsid protein. **g** Phylogeny of viruses in the family *Phenuiviridae* based on amino acid sequence of nucleoprotein. **h** Phylogeny of viruses in the family *Totiviridae* based on amino acid sequence of RdRp protein. Viruses detected in this study are marked by red solid circles for ticks in clade 1 and blue solid circles for ticks in clade 2

The viruses shared by both clades had relatively high prevalences compared to the clade-specific viruses, with no significant difference in positive rate of each virus between the two clades (Fig. 4a). Among them, *Hepelivirales* sp. in the order *Hepelivirales* had the highest positive rate both in clade 1 (54.00%, 95% CI 44.28–63.72%) and clade 2 (38.89%, 95% CI 23.05–54.73%). In the phylogenetic tree, *Hepelivirales* sp. were mixed with *Hepelivirales* sp. detected in *H. longicornis* ticks from various areas of China (Fig. 4b) [15], showing extensively distribution of the virus in the tick species. *Cheeloo Jingmen-like virus,* a segmented virus in the family *Flaviviridae* was the second prevalent virus (Fig. 4a). Its complete putative glycoprotein (VP1a) and VP1b protein shared 91.20–100% and 92.12–100% aa identity, respectively, with their closest *Alongshan virus* (GenBank accession no. MZ676705) from *H. longicornis* (Supplementary Fig. 2). Phylogenetic analysis based on putative VP1b protein revealed that the virus clustered with a *Alongshan virus* in the Jingmenvirus group and was distinct from other members (Fig. 4c). Two virus species in the *Peribunyaviridae* family, *Henan tick virus* and *Shanxi tick virus* that had been previously reported in *H. longicornis* respectively from Henan and Shanxi provinces of China [11], were also detected in both clads of *H. longicornis* (Fig. 4d).

*Hubei sobemo-like virus 15* in the *Solemoviridae* family had a higher positive rate of 25.74% (95% CI 18.43–33.05%) and showed 97.27–98.84% nt identities and 99.16–100% aa identities with the known strain detected in ticks (GenBank accession no. NC_032208) [40]. In addition, a novel virus in the same family, which we called *Cheeloo tick virus 3*, was distinct from *Hubei sobemo-like virus 15* and formed a separate branch in the phylogenetic tree (Fig. 4e). They had only 52.15% aa identity of RdRp with the closest species, *Lone star tick associated virus-1* in *Amblyomma americanum* ticks from USA (GenBank accession no. MF962658). Segments of two distinct *Nodaviridae*-like viruses, which we called *Cheeloo noda-like virus* 2 and 3, were detected in both clades. The phylogenetic analysis based on coat protein revealed that either *Cheeloo noda-like virus* 2 or *Cheeloo noda-like virus* 3 formed a branch distinct from all known virus species in the *Nodaviridae* family (Fig. 4f). A novel segmented virus named *Cheeloo phenui-like virus* in the *Phenuiviridae* family was in a separate branch of the phylogenetic tree (Fig. 4g), only with 38.97–41.18% aa identity of nucleoprotein with the closest *Lone star bandavirus* identified in *A. americanum* ticks from USA (GenBank accession no. YP_008003509) [41]. Another new virus species named *Cheeloo toti-like virus 2* was closely related to but distinguishing from *Hubei toti-like virus 24* previously detected in ticks from Hubei Province of China (GenBank accession no. NC_032938) [40], with only 41.29–41.41% aa identities of RdRp (Fig. 4h).

### Clade 1-specific viruses

**Fig. 5:**
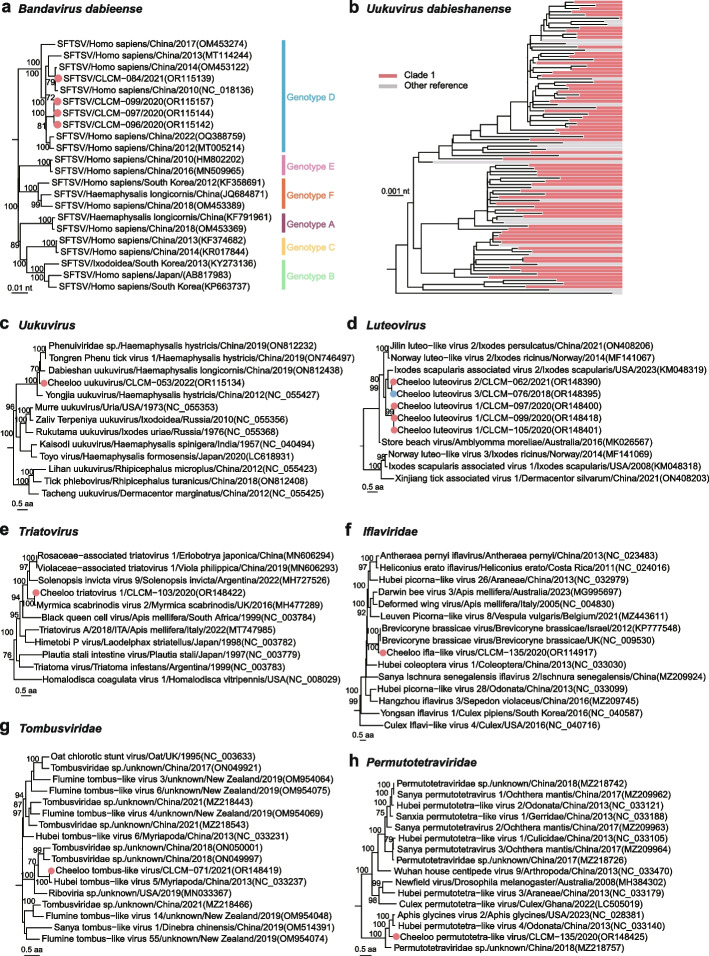
Phylogenetic analysis of clade 1-specific viruses. **a** Phylogenetic tree of *Bandavirus dabieense* based on nucleotide sequences of RdRp gene. **b** Phylogeny of *Uukuvirus dabieshanense* based on nucleotide sequence of RNA-dependent RNA polymerase (RdRp) gene. The red strips indicate tick samples from clade 1 and the gray strips are reference sequences. **c** Phylogeny of viruses in the genus of *Uukuvirus* based on amino acid sequence of RdRp protein. **d** Phylogeny of viruses in the genus *Luteovirus* based on amino acid sequence of RdRp protein. **e** Phylogeny of viruses in the genus *Triatovirus* based on amino acid sequence of RdRp protein. **f** Phylogeny of viruses in the genus *Iflaviridae* based on amino acid sequence of RdRp protein. **g** Phylogeny of viruses in the family *Tombusviridae* based on amino acid sequence of RdRp protein. **h** Phylogeny of viruses in the family *Permutotetraviridae* based on amino acid sequence of RdRp protein. Viruses in this study are marked by solid circles

An important clade 1-specific virus is *Bandavirus dabieense*, the causative agent of an emerging tick-borne disease, severe fever with thrombocytopenia syndrome (SFTS) [12]. The virus was only identified in four tick samples of clade 1 (Fig. 5a, Supplementary Fig. 3). To validate the results of *Bandavirus dabieense* found in the meta-transcriptome analysis, we tested all remaining samples available after meta-transcriptomic sequencing with specific primers, and the four positive tick samples were confirmed by real time RT-PCR (Supplementary Fig. 4). Phylogenetic analysis based on the L segment showed that the *Bandavirus dabieense* in tick clade 1 was clustered in genotype D (Fig. 5a), which is currently circulating in humans of China. The association of the emerging virus and the genetic clade of its tick vector deserve further investigation to better understand vector-pathogen interaction of SFTS. Another clade 1-specific virus is *Uukuvirus dabieshanense* in the family *Phenuiviridae*, which was detected in 71% ticks in clade 1, but never in clade 2. The phylogenetic analysis based on either L (Fig. 5b) or S (Supplementary Fig. 5) gene indicated the virus in this study was mixed with those presented in various tick species from different regions of China [42–45], indicating its extensive circulation and adaptation. On the other hand, the specificity to clade 1 *H. longicornis* suggests the genetic and evolutionary impacts of ticks on the virus infection, which deserves further investigation.

In addition, other 18 viruses specific to clade 1 were identified, of which 11 were known viruses, mainly arthropod-borne viruses (Supplementary Fig. 6). The other seven newly identified viruses were detected in only one or three samples. *Cheeloo uukuvirus* was in one sample and most close to *Uukuvirus dabieshanense* (Fig. 5c) with 70.09% aa similarity of RdRp domain [11]. *Cheeloo luteovirus 1* was in three samples and most closely related to *Ixodes scapularis-associated virus 2* (Fig. 5d) with 71.81% aa identity of RdRp protein [46]. *Cheeloo triatovirus 1* and *Cheeloo ifla-like virus* were two new members of genera *Triatovirus* and *Iflavirus* (Fig. 5e, f), the closest viruses of which were detected from red ant (*Myrmica scabrinodis*) and aphid (*Brevicoryne brassicae*) with 87.37% and 56.98% aa homology of RdRp protein, respectively from UK [47, 48], suggesting the two tick-related viruses might be obtained through cross-transmission from other arthropod on the same vegetation and adapted to the ticks of clade 1 during long evolution. The other two viruses, *Cheeloo tombus-like virus* and *Cheeloo permutotetra-like virus*, were new members of the families *Tombusviridae* and *Permutotetraviridae* (Fig. 5g, h) and exhibited 87.71% aa identity of RdRp with the closest *Hubei tombus-like virus 5* and 58.16% aa identity of RdRp protein with the closest *Hubei permutotetra-like virus 4* from invertebrates [40], indicating the viruses’ possible evolutionary history.

### Half of clade 2-specific viruses were aquatic-animal-associated

As described above, *H. longicornis* in clade 2 mainly distributed in Jiaodong Peninsula and northern plain regions of Shandong Province (Supplementary Fig. 7). Five known arthropod-associated viruses and three newly identified viruses, *Cheeloo triatovirus 2*, *Cheeloo luteovirus 3*, and *Cheeloo toti-like virus 1*, which were phylogenetically distinct from all known viruses, were only detected in clade 2 (Supplementary Fig. 8 and Fig. 5d).

**Fig. 6:**
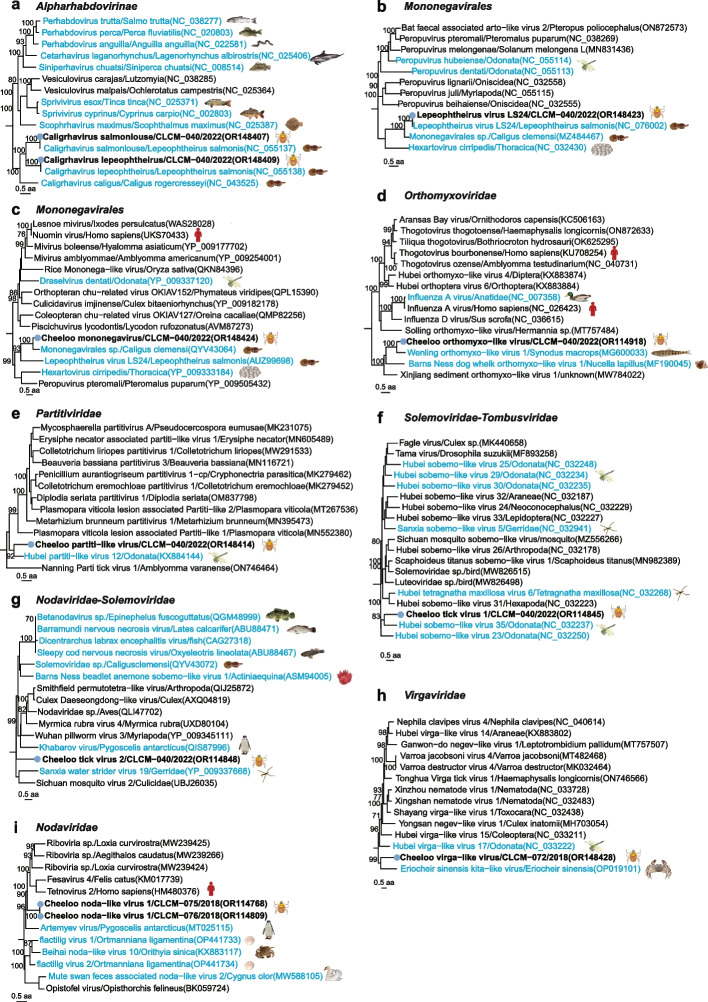
Phylogenetic analysis of aquatic-animal-associated viruses in clade 2. **a** Phylogeny of viruses in the subfamily of *Alpharhabdovirinae* based on amino acid sequence of RNA-dependent RNA polymerase (RdRp) protein. **b** Phylogeny of viruses in the order of *Mononegavirales* based on amino acid sequence of RdRp protein. **c** Phylogeny of viruses in the order of *Mononegavirales* based on amino acid sequence of glycoprotein. **d** Phylogeny of viruses in the family of *Orthomyxoviridae* based on amino acid sequence of PB1 protein. **e** Phylogeny of viruses in the family of *Partitiviridae* based on amino acid sequence of RdRp protein. **f** Phylogeny of viruses between the family *Solemoviridae* and *Tombusviridae* based on amino acid sequence of RdRp protein. **g** Phylogeny of viruses between the family *Nodaviridae* and *Solemoviridae* based on amino acid sequence of coat protein. **h** Phylogeny of viruses in the family of *Virgaviridae* based on amino acid sequence of RdRp protein. **i** Phylogeny of viruses in the family of *Nodaviridae* based on amino acid sequence of RdRp protein. Viruses in this study are marked in bold. The animal hosts of viruses are labeled with cartoon images

Interestingly, ten of the 18 clade 2-specific viruses were known or genetically close to the aquatic-animal-associated viruses, which we describe in more detail below. The RdRp protein of *Caligrhavirus salmonlouse* and *Caligrhavirus lepeophtheirus* had 94.5% and 93.26% similarities with the known viruses in the family *Rhabdoviridae* (Fig. 6a), and that of *Lepeophtheirus virus* in the order *Mononegavirales* were shared 99.77% identity with the known species that had been detected in sea lice (*Lepeophtheirus salmonis*) [49] (Fig. 6b). *Cheeloo mononegavirus* was a novel species most closely related to *Mononegavirales* sp. also from sea louse *Caligus clemensi* (GenBank accession no. QYV43064) with 42.97% aa identity of glycoprotein (Fig. 6c). *Cheeloo orthomyxo-like virus* was genetically close to *Wenling orthomyxo-like virus 1* (GenBank accession no. MG600033) from lizard fish (*Synodus macrops*) in China [35] with a 50.53% aa identity of RdRp protein (Fig. 6d). *Cheeloo partiti-like virus* and *Cheeloo tick virus 1* were respectively closest to *Hubei partiti-ike virus 12* (GenBank accession no. KX884144) related to the family *Partitiviridae* with 50.79% identity of RdRp protein and *Hubei sobemo-like virus 35* (GenBank accession no. NC_032237) related to the genus *Sobemovirus* with 41.24% identity of RdRp protein (Fig. 6e, f) [40], both from dragonfly (*Odonata*), which must rely on freshwater to lay eggs and hatch. *Cheeloo tick virus 2* only had 22.46% homology of coat protein with genetically closest *Sanxia water strider virus* 19 (GenBank accession no. YP009337668) (Fig. 6g) initially identified in water strider (*Gerridae*) [40], which live on the water surface. *Cheeloo virga-like virus* was clustered with *Eriocheir sinensis kita-like virus* (GenBank accession no. OP019101) in the family *Kitaviridae* (Fig. 6h) previously recognized in Chinese crab (*Eriocheir sinensis*), with 19.41% identity of RdRp protein. *Cheeloo noda-like virus 1* in the family *Nodaviridae* was close to *Artemyev virus* (GenBank accession no. MT025115) from penguin (*Pygoscelis antarcticus*) with 30.84% aa identity of RdRp (Fig. 6i) [39].

**Fig. 7:**
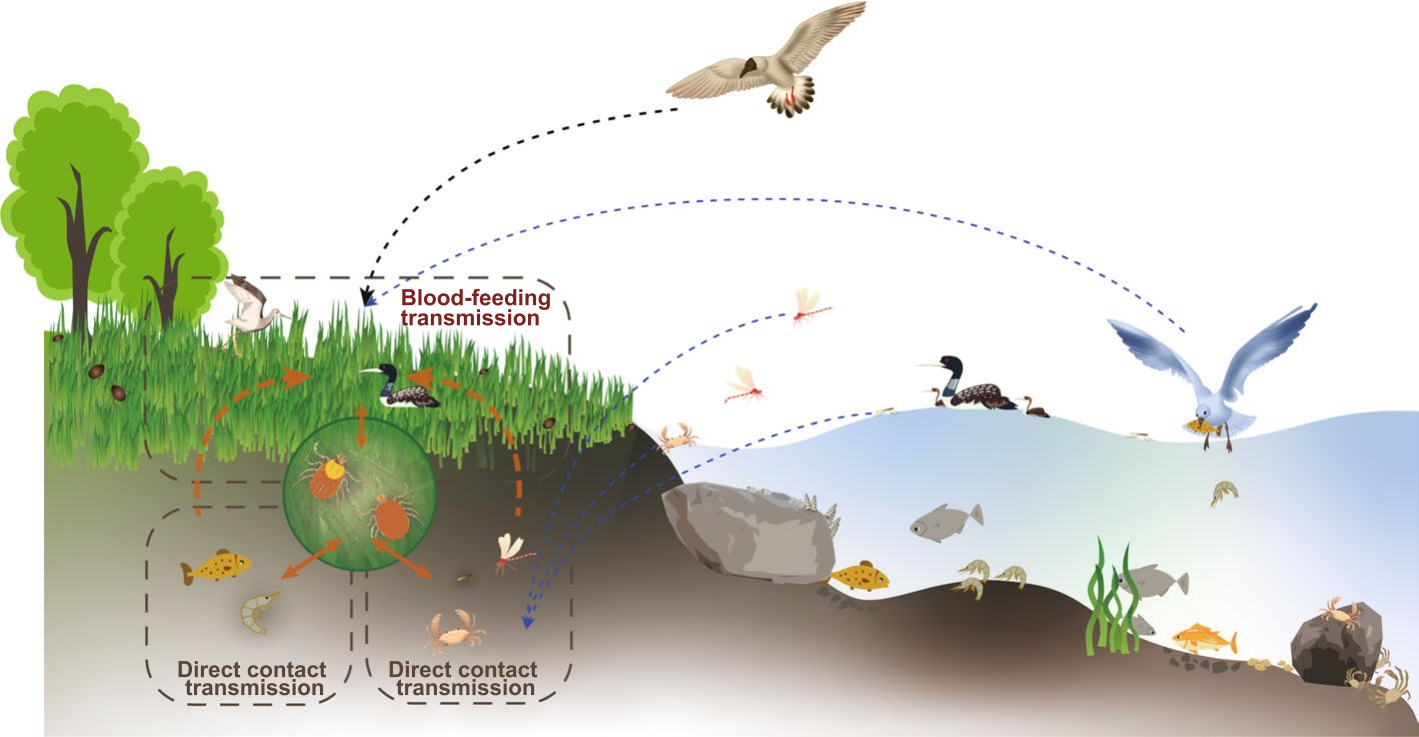
Ecological schematic diagram of *H. longicornis* in clade 2. The red arrows indicate the path of the virus spreading

The aquatic animal-associated viruses that appear only in ticks of clade 2 might originate from complex ecological environments and result from long-term co-evolution with ticks (Fig. 7). First, these aquatic-animal-associated viruses carried by ticks might have been acquired through direct contact of ticks with aquatic animals such as crabs as well as surface insects including dragonflies and water strider, which crawl or move to the nearby grassland. Secondly, these viruses might also be acquired by ticks exposed to the aquatic animals, such as fish and shrimp, captured and brought to land by waterfowls. Moreover, waterfowls, especially migratory birds, may play a role in spreading these viruses. On the one hand, aquatic-animal-associated viruses carried by waterfowls might infect ticks through biting and bloodsucking. On the other hand, it is possible that migratory birds brought infected ticks from other locations to the survey site, although these aquatic-animal-associated viruses identified in this study were not reported in ticks from other places (Fig. 7). To validate this hypothesis, surveillance of viruses in wildfowls and their parasitic ticks should be enhanced.

## Discussion

Previous meta-transcriptomic investigations targeting tick viromes have discovered a growing number of divergent viruses around the world [11, 15, 39, 43–46, 50, 51]. However, there has been no study concerning the role of both evolutionary and ecological elements in diversity of tick-associated viruses. In this study, we focused on the highly invasive and extensive tick species, *H. longicornis*, and performed meta-transcriptomic sequencing of ticks collected across Shandong Province of China. Because *H. longicornis* is the predominate tick species in the province, where there is an area of 158,000 square kilometers, this gives us a chance to investigate the viromic diversity in relation to the genetic evolution of *H. longicornis* population and the ecological factors. From the 136 tick samples, we discovered 508 RNA viruses belonging to 48 viral species, 22 of which have never been reported before, further proving the great diversity of viruses in *H. longicornis*. These findings of virome investigation will contribute to discovery of new pathogenic viruses from the etiological aspect [52] and suggest that some of these may pose a potential threat to public and veterinary health, hence requiring intensive surveillance [53].

*H. longicornis* ticks in this study have two distinct genetic clades according to evolutionary and comparative genomic analyses, one mainly clustering in mountain areas and the other in plain. The multivariate analysis reveals that the different distribution is significantly associated with three ecological factors. Our findings suggest that the ticks in each clade have found their suitable habitats through long-term adaptive evolution and movement restriction in the specific ecological landscape. In addition, the role of animal hosts should not be ignored considering its high adaptability to environments and a broad range of hosts involving domestic and wild animals. The virome diversity was significantly lower in fed than unfed *H. longicornis* ticks which might be attributable to the inhibition on virus replication of antimicrobial peptides from the host blood or to activated complement that have been known to play an important role in immune responses against bacteria and viruses in ticks [54]. In view of the discriminating impacts of blood meal from different hosts on microbiome diversity of ticks [55], further experimental studies feeding on different animals to validate the influence on virome richness and composition of blood meals from different hosts, and ultimately to assess potential vector competency of *H. longicornis* at diverse ecological habitats.

We find that the viruses shared by both clades of *H. longicornis* always have relatively high positive rates in comparison to the clade-specific viral species. This finding together with the previous reports indicate that these viruses have evolved into generalists in the ticks to better tolerate or adapt to environmental changes [11, 56]. Therefore, the vector competence of *H. longicornis* for transmitting these viruses deserve further investigation. Considering that *Henan tick virus* and *Shanxi tick virus 2* are often detected in clade 1 and 2 ticks, and that *Burana orthonairovirus* and *Songling virus* in the family *Peribunyaviridae* are pathogenic to humans, their public health significance should not be ignored and continuous surveillance should be strengthened.

Strikingly, *Bandavirus dabieense*, the causative agent of human SFTS, were only detected in ticks of clade 1, suggesting evolutionary characteristics of ticks might determine their capacity as vectors. Although our previous experimental study proved that *H. longicornis* is a competent vector to transmit this virus in both transovarial and transstadial modes [57], we do not know if the ticks used for the study belong to clade 1 due to lack of such genetic classification at that time. Further experiments using *H. longicornis* ticks in different genetic clades can clarify this issue. Another interesting finding is that several aquatic-animal-associated viruses only present in ticks of clade 2. Although *H. longicornis* is known feed on a wide variety of animal hosts [3], there has been no evidence that the tick species can feed on aquatic animals [5]. We assume that these ticks most likely get such viruses from the same ecological landscape shared by the aquatic animals, rather than directly from these animals. The relative lower elevation of breeding areas (Fig. 2b, d) might have provided the chance for the ticks in clade 2 to contact with the aquatic animals. Furthermore, our findings reveal that these tick-borne aquatic-animal-associated viruses genetically close to but not identical to the known aquatic viruses, indicating the evolution of the viruses driven by the possible biological cycles mentioned in Fig. 7.

One limitation of our study is the pooling of multiple ticks with the same sex, sampling sites and blood-feeding status into one sequencing library, which means that the virome in an individual tick cannot be tested, and rare or less abundant viruses are possibly missed. Furthermore, the use of pooling sample tests cannot accurately reflect the prevalence of viruses among ticks.

In summary, our findings not only further prove the high viromic diversity of *H. longicornis* but also reveals its internal genetic evolution and external ecological landscape shaped viral composition and prevalence. On the other hand, the obvious geographical clustering of both tick clade and clade-specific viruses highlights the evident ability of *H. longicornis* to adapt to divergent ecological environments and animal hosts. The findings of current study provide the new foundation for promoting the studies on virus-vector-ecology interaction and eventually for evaluating the risk of *H. longicornis* for transmitting the viruses to humans and animals.

### Supplementary Information

**Additional file 1: Supplementary Table 1.** Basic information of each library for sequencing in this study. **Supplementary Table 2.** Viral sequences of this study deposited in GenBank. **Supplementary Table 3.** The prevalence with 95% confidence interval (CI) of viruses in two clades. **Supplementary Fig. 1.** The virome diversity. **Supplementary Fig. 2.** Phylogenetic analysis of Cheeloo Jingmen-like virus based on glycoprotein. **Supplementary Fig. 3.** Phylogenetic analysis of Bandavirus dabieense. **Supplementary Fig. 4.** Validation of Bandavirus dabieense. **Supplementary Fig. 5.** Phylogeny of Uukuvirus dabieshanense based on nucleotide sequence of S gene. **Supplementary Fig. 6.** Phylogenic analysis of known viruses in clade 1. **Supplementary Fig. 7.** The geographical distribution of H. longicornis in clade 2. **Supplementary Fig. 8.** Phylogenic analysis of arthropod-associated viruses in clade 2.

## Data Availability

The sequencing data are available from the NCBI Sequence Read Archive (SRA) (SRR24768944–SRR24769079) under Bioproject PRJNA976632 (temporarily available from the Reviewer link: https://dataview.ncbi.nlm.nih.gov/object/PRJNA976632?reviewer=md2civ8mh835gldnulkc3pu2k1), and the assembled virus sequences are available as of the date of publication in the GenBank with the accession no. OR114691–OR115157 and OR148387–OR148428.
