# Supplementary material for: Virome diversity shaped by genetic evolution and ecological landscape of Haemaphysalis longicornis
Source: Microbiome. 2024 Feb 21;12:35. doi: 10.1186/s40168-024-01753-9 (PMC10880243; doi:10.1186/s40168-024-01753-9)
Supplement: Supplementary file 2 — Additional file 1: Supplementary Table 1. Basic information of each library for sequencing in this study. Supplementary Table 2. Viral sequences of this study deposited in GenBank. Supplementary Table 3. The prevalence with 95% confidence interval (CI) of viruses in two clades. Supplementary Fig. 1. The virome diversity. Supplementary Fig. 2. Phylogenetic analysis of Cheeloo Jingmen-like virus based on glycoprotein. Supplementary Fig. 3. Phylogenetic analysis of Bandavirus dabieense. Supplementary Fig. 4. Validation of Bandavirus dabieense. Supplementary Fig. 5. Phylogeny of Uukuvirus dabieshanense based on nucleotide sequence of S gene. Supplementary Fig. 6. Phylogenic analysis of known viruses in clade 1. Supplementary Fig. 7. The geographical distribution of H. longicornis in clade 2. Supplementary Fig. 8. Phylogenic analysis of arthropod-associated viruses in clade 2. [file 40168_2024_1753_MOESM1_ESM.pdf]

Additional file 1

Supplementary Table 1. Basic information of each library for sequencing in this study.. 2

Supplementary Table 2. Viral sequences of this study deposited in GenBank 6

Supplementary Table 3. The prevalence with 95% confidence interval (CI) of viruses in two clades 17

Supplementary Figure 1. The virome diversity. 19

Supplementary Figure 2. Phylogenetic analysis of *Cheeloo Jingmen-like virus* based on glycoprotein.. 20

Supplementary Figure 3. Phylogenetic analysis of *Bandavirus dabieense* 21

Supplementary Figure 4. Validation of *Bandavirus dabieense* 22

Supplementary Figure 5. Phylogeny of *Uukuvirus dabieshanense* based on nucleotide sequence of S gene 23

Supplementary Figure 6. Phylogenic analysis of known viruses in clade 1 24

Supplementary Figure 7. The geographical distribution of *H. longicornis* in clade 2 26

Supplementary Figure 8. Phylogenic analysis of arthropod-associated viruses in clade 2 27

Supplementary Table 1. Basic information of each library for sequencing in this study

City	Library ID	Gender	Clean reads (paired-end)	Percentage of viral reads (%)	Number of viral contigs
Binzhou	CLCM-045	Female	5288224	0.14	24
Binzhou	CLCM-046	Female	10799218	0.04	97
Dezhou	CLCM-038	Female	4705831	3.19	195
Dezhou	CLCM-039	Female	4819487	0.1	231
Dezhou	CLCM-040	Female	6812406	0.22	88
Dezhou	CLCM-041	Female	6488970	3.22	90
Dezhou	CLCM-042	Female	5847981	1.91	213
Dezhou	CLCM-077	Female	5151450	0.1	301
Dezhou	CLCM-078	Female	12251505	0.44	38
Dezhou	CLCM-079	Male	5510662	0.16	53
Dezhou	CLCM-080	Male	4904866	0.16	243
Dezhou	CLCM-081	Male	6123040	0.72	214
Dongying	CLCM-049	Female	5738269	0.13	316
Dongying	CLCM-050	Female	4211284	0.13	217
Heze	CLCM-061	Female	19051195	0.13	410
Liaocheng	CLCM-043	Female	2297627	0.13	71
Liaocheng	CLCM-044	Female	7290104	0.09	26
Linyi	CLCM-030	Female	4098444	0.26	186
Linyi	CLCM-031	Male	3851969	0.52	108
Linyi	CLCM-032	Male	4342675	0.18	95
Linyi	CLCM-033	Female	5159745	1.01	156
Linyi	CLCM-034	Male	4127280	0.49	368
Linyi	CLCM-035	Male	6151224	0.86	69
Linyi	CLCM-036	Female	1239834	2.76	71
Linyi	CLCM-037	Male	3795544	2.57	124
Linyi	CLCM-060	Male	6857584	1.9	125
Linyi	CLCM-062	Male	10043743	0.04	267
Linyi	CLCM-063	Female	1372143	2.41	399
Linyi	CLCM-064	Female	735095	1.55	215
Linyi	CLCM-065	Female	1218845	10.72	182
Linyi	CLCM-066	Male	528021	0.65	166
Linyi	CLCM-067	Male	1660434	1.65	110
Linyi	CLCM-068	Male	704566	0.24	189
Linyi	CLCM-069	Male	7480092	0.11	242
Linyi	CLCM-070	Male	5975382	0.32	261
Linyi	CLCM-071	Male	6925527	0.21	61
Qingdao	CLCM-002	Female	3178122	0.06	132
Qingdao	CLCM-003	Female	4465011	1.72	137
Qingdao	CLCM-004	Female	4138792	0.09	313
Qingdao	CLCM-011	Male	2778495	0.69	369
Qingdao	CLCM-012	Female	1745534	0.17	68

Qingdao	CLCM-014	Female	5122104	0.09	101
Qingdao	CLCM-072	Female	1901458	12.23	47
Qingdao	CLCM-073	Female	1834741	14.05	80
Qingdao	CLCM-074	Female	1607066	11.74	175
Qingdao	CLCM-075	Male	12715777	0.22	155
Qingdao	CLCM-076	Female	2556156	0.49	106
Rizhao	CLCM-056	Female	6877162	1.32	92
Rizhao	CLCM-057	Male	4820939	1.59	205
Rizhao	CLCM-058	Female	4361288	2.15	272
Rizhao	CLCM-059	Female	3862360	0.37	89
Taian	CLCM-013	Female	4860310	0.14	173
Taian	CLCM-025	Male	774168	0.11	237
Taian	CLCM-027	Female	4869083	0.16	60
Taian	CLCM-028	Male	5322941	0.15	58
Taian	CLCM-082	Male	5766341	1.07	83
Taian	CLCM-083	Male	5157645	2.28	62
Taian	CLCM-084	Male	4984282	1.46	79
Taian	CLCM-085	Female	4073552	3.62	95
Taian	CLCM-086	Female	5162000	1.96	68
Taian	CLCM-087	Female	4900035	0.3	97
Taian	CLCM-088	Male	2324329	0.55	153
Taian	CLCM-089	Male	2345085	4.81	74
Taian	CLCM-090	Female	3100545	6.5	44
Taian	CLCM-091	Female	4009955	1.64	33
Taian	CLCM-092	Male	5077953	0.68	32
Taian	CLCM-093	Female	5231174	0.37	59
Taian	CLCM-094	Male	4815873	1.97	59
Taian	CLCM-095	Female	10385738	0.79	170
Weifang	CLCM-096	Female	5301457	15.64	128
Weifang	CLCM-097	Female	14903015	2.29	473
Weifang	CLCM-098	Female	677346	28.56	245
Weifang	CLCM-099	Male	14779371	6.22	172
Weifang	CLCM-100	Female	2017116	8.35	219
Weifang	CLCM-101	Female	1502027	4.57	331
Weifang	CLCM-102	Female	2047631	0.1	176
Weifang	CLCM-103	Female	2236208	8.26	45
Weifang	CLCM-104	Male	1133989	9.23	84
Weifang	CLCM-105	Male	3920636	27.26	268
Weifang	CLCM-106	Male	1260194	10.66	324
Weifang	CLCM-107	Male	1660035	43.09	154
Weifang	CLCM-108	Female	647187	17.88	112
Weihai	CLCM-109	Female	3496005	4.23	131
Weihai	CLCM-110	Female	2828001	9.57	135
Weihai	CLCM-111	Male	7109511	14.8	167

Weihai	CLCM-112	Female	8774200	0.18	111
Weihai	CLCM-113	Female	2182353	8.29	104
Weihai	CLCM-114	Female	1999032	14.23	157
Weihai	CLCM-115	Female	3424344	5.81	180
Weihai	CLCM-116	Male	1413015	10.34	147
Weihai	CLCM-117	Male	2193594	18.44	170
Weihai	CLCM-118	Female	2773798	5.64	152
Weihai	CLCM-119	Female	2920274	15.26	111
Weihai	CLCM-120	Female	3982485	8.32	92
Yantai	CLCM-005	Female	4319872	0.06	157
Yantai	CLCM-006	Female	6115739	0.08	355
Yantai	CLCM-008	Male	6103196	0.1	558
Yantai	CLCM-009	Female	760505	0.04	197
Yantai	CLCM-010	Female	1539089	0.98	868
Yantai	CLCM-051	Female	5402570	0.14	295
Yantai	CLCM-052	Female	5379912	0.71	260
Yantai	CLCM-053	Male	9080582	0.13	358
Yantai	CLCM-054	Female	4371173	0.94	390
Yantai	CLCM-055	Male	5071664	0.22	187
Yantai	CLCM-121	Male	1436909	37.69	454
Yantai	CLCM-122	Male	472189	13.86	233
Yantai	CLCM-123	Female	1120365	27.05	406
Yantai	CLCM-124	Female	831318	17.74	205
Zaozhuang	CLCM-001	Female	418591	0.1	125
Zaozhuang	CLCM-007	Male	2473822	0.11	130
Zaozhuang	CLCM-022	Female	5687229	3.5	143
Zaozhuang	CLCM-023	Female	1017857	0.16	208
Zaozhuang	CLCM-024	Female	543060	0.06	387
Zaozhuang	CLCM-026	Female	4515718	0.11	329
Zaozhuang	CLCM-029	Male	4925965	0.13	574
Zibo	CLCM-015	Female	4751640	0.1	543
Zibo	CLCM-016	Male	1470565	2.19	614
Zibo	CLCM-017	Female	595378	2.38	489
Zibo	CLCM-018	Female	5543297	0.1	392
Zibo	CLCM-019	Female	7117265	0.93	628
Zibo	CLCM-020	Male	710429	0.12	230
Zibo	CLCM-021	Male	1128827	1.21	137
Zibo	CLCM-047	Male	3788309	0.14	258
Zibo	CLCM-048	Female	5282059	0.46	242
Zibo	CLCM-125	Male	510126	21.46	50
Zibo	CLCM-126	Female	762504	9.4	53
Zibo	CLCM-127	Male	320389	9.92	45
Zibo	CLCM-128	Male	362475	38.79	77
Zibo	CLCM-129	Female	358108	18.44	60

Zibo	CLCM-130	Male	256014	18.38	59
Zibo	CLCM-131	Female	1281306	5.46	169
Zibo	CLCM-132	Male	981293	9.9	209
Zibo	CLCM-133	Female	1736962	6.27	346
Zibo	CLCM-134	Male	1576628	1.33	118
Zibo	CLCM-135	Female	2498694	6.5	263
Zibo	CLCM-136	Male	2233122	12.1	224

Supplementary Table 2. Viral sequences of this study deposited in GenBank

Accession no.	Sample ID	Species
OR114691	CLCM-072	*Hepelivirales* sp.
OR114692	CLCM-073	*Hepelivirales* sp.
OR114693	CLCM-074	*Hepelivirales* sp.
OR114694	CLCM-010	*Hepelivirales* sp.
OR114695	CLCM-016	*Hepelivirales* sp.
OR114696	CLCM-017	*Hepelivirales* sp.
OR114697	CLCM-018	*Hepelivirales* sp.
OR114698	CLCM-019	*Hepelivirales* sp.
OR114699	CLCM-022	*Hepelivirales* sp.
OR114700	CLCM-023	*Hepelivirales* sp.
OR114701	CLCM-034	*Hepelivirales* sp.
OR114702	CLCM-035	*Hepelivirales* sp.
OR114703	CLCM-036	*Hepelivirales* sp.
OR114704	CLCM-037	*Hepelivirales* sp.
OR114705	CLCM-042	*Hepelivirales* sp.
OR114706	CLCM-052	*Hepelivirales* sp.
OR114707	CLCM-056	*Hepelivirales* sp.
OR114708	CLCM-057	*Hepelivirales* sp.
OR114709	CLCM-058	*Hepelivirales* sp.
OR114710	CLCM-082	*Hepelivirales* sp.
OR114711	CLCM-083	*Hepelivirales* sp.
OR114712	CLCM-084	*Hepelivirales* sp.
OR114713	CLCM-085	*Hepelivirales* sp.
OR114714	CLCM-086	*Hepelivirales* sp.
OR114715	CLCM-087	*Hepelivirales* sp.
OR114716	CLCM-088	*Hepelivirales* sp.
OR114717	CLCM-089	*Hepelivirales* sp.
OR114718	CLCM-090	*Hepelivirales* sp.
OR114719	CLCM-091	*Hepelivirales* sp.
OR114720	CLCM-094	*Hepelivirales* sp.
OR114721	CLCM-098	*Hepelivirales* sp.
OR114722	CLCM-099	*Hepelivirales* sp.
OR114723	CLCM-100	*Hepelivirales* sp.
OR114724	CLCM-102	*Hepelivirales* sp.
OR114725	CLCM-104	*Hepelivirales* sp.
OR114726	CLCM-105	*Hepelivirales* sp.
OR114727	CLCM-106	*Hepelivirales* sp.
OR114728	CLCM-107	*Hepelivirales* sp.
OR114729	CLCM-108	*Hepelivirales* sp.
OR114730	CLCM-109	*Hepelivirales* sp.
OR114731	CLCM-110	*Hepelivirales* sp.
OR114732	CLCM-111	*Hepelivirales* sp.
OR114733	CLCM-112	*Hepelivirales* sp.
OR114734	CLCM-113	*Hepelivirales* sp.
OR114735	CLCM-114	*Hepelivirales* sp.
OR114736	CLCM-115	*Hepelivirales* sp.
OR114737	CLCM-116	*Hepelivirales* sp.

OR114738	CLCM-117	*Hepelivirales* sp.
OR114739	CLCM-118	*Hepelivirales* sp.
OR114740	CLCM-119	*Hepelivirales* sp.
OR114741	CLCM-120	*Hepelivirales* sp.
OR114742	CLCM-121	*Hepelivirales* sp.
OR114743	CLCM-126	*Hepelivirales* sp.
OR114744	CLCM-128	*Hepelivirales* sp.
OR114745	CLCM-129	*Hepelivirales* sp.
OR114746	CLCM-131	*Hepelivirales* sp.
OR114747	CLCM-132	*Hepelivirales* sp.
OR114748	CLCM-133	*Hepelivirales* sp.
OR114749	CLCM-135	*Hepelivirales* sp.
OR114750	CLCM-136	*Hepelivirales* sp.
OR114751	CLCM-014	*Hepelivirales* sp.
OR114752	CLCM-033	*Hepelivirales* sp.
OR114753	CLCM-005	*Hepelivirales* sp.
OR114754	CLCM-006	*Hepelivirales* sp.
OR114755	CLCM-096	*Hepelivirales* sp.
OR114756	CLCM-097	*Hepelivirales* sp.
OR114757	CLCM-101	*Hepelivirales* sp.
OR114758	CLCM-103	*Hepelivirales* sp.
OR114759	CLCM-041	*Huangpi orthonairovirus*
OR114760	CLCM-041	*Huangpi orthonairovirus*
OR114761	CLCM-041	*Huangpi orthonairovirus*
OR114762	CLCM-042	*Huangpi orthonairovirus*
OR114763	CLCM-042	*Huangpi orthonairovirus*
OR114764	CLCM-042	*Huangpi orthonairovirus*
OR114765	CLCM-119	*Huangpi orthonairovirus*
OR114766	CLCM-119	*Huangpi orthonairovirus*
OR114767	CLCM-119	*Huangpi orthonairovirus*
OR114768	CLCM-075	*Cheeloo noda-like virus 1*
OR114769	CLCM-067	*Cheeloo noda-like virus 2*
OR114770	CLCM-073	*Cheeloo noda-like virus 2*
OR114771	CLCM-012	*Cheeloo noda-like virus 2*
OR114772	CLCM-022	*Cheeloo noda-like virus 2*
OR114773	CLCM-031	*Cheeloo noda-like virus 2*
OR114774	CLCM-036	*Cheeloo noda-like virus 2*
OR114775	CLCM-037	*Cheeloo noda-like virus 2*
OR114776	CLCM-038	*Cheeloo noda-like virus 2*
OR114777	CLCM-056	*Cheeloo noda-like virus 2*
OR114778	CLCM-057	*Cheeloo noda-like virus 2*
OR114779	CLCM-092	*Cheeloo noda-like virus 2*
OR114780	CLCM-096	*Cheeloo noda-like virus 2*
OR114781	CLCM-097	*Cheeloo noda-like virus 2*
OR114782	CLCM-098	*Cheeloo noda-like virus 2*
OR114783	CLCM-099	*Cheeloo noda-like virus 2*
OR114784	CLCM-106	*Cheeloo noda-like virus 2*
OR114785	CLCM-111	*Cheeloo noda-like virus 2*
OR114786	CLCM-117	*Cheeloo noda-like virus 2*

OR114787	CLCM-118	*Cheeloo noda-like virus 2*
OR114788	CLCM-119	*Cheeloo noda-like virus 2*
OR114789	CLCM-123	*Cheeloo noda-like virus 2*
OR114790	CLCM-127	*Cheeloo noda-like virus 2*
OR114791	CLCM-128	*Cheeloo noda-like virus 2*
OR114792	CLCM-130	*Cheeloo noda-like virus 2*
OR114793	CLCM-132	*Cheeloo noda-like virus 2*
OR114794	CLCM-134	*Cheeloo noda-like virus 2*
OR114795	CLCM-135	*Cheeloo noda-like virus 2*
OR114796	CLCM-136	*Cheeloo noda-like virus 2*
OR114797	CLCM-062	*Cheeloo noda-like virus 3*
OR114798	CLCM-071	*Cheeloo noda-like virus 3*
OR114799	CLCM-072	*Cheeloo noda-like virus 3*
OR114800	CLCM-075	*Cheeloo noda-like virus 3*
OR114801	CLCM-030	*Cheeloo noda-like virus 3*
OR114802	CLCM-039	*Cheeloo noda-like virus 3*
OR114803	CLCM-053	*Cheeloo noda-like virus 3*
OR114804	CLCM-107	*Cheeloo noda-like virus 3*
OR114805	CLCM-115	*Cheeloo noda-like virus 3*
OR114806	CLCM-120	*Cheeloo noda-like virus 3*
OR114807	CLCM-131	*Cheeloo noda-like virus 3*
OR114808	CLCM-133	*Cheeloo noda-like virus 3*
OR114809	CLCM-076	*Cheeloo noda-like virus 1*
OR114810	CLCM-065	*Cheeloo noda-like virus 2*
OR114811	CLCM-109	*Cheeloo noda-like virus 2*
OR114812	CLCM-065	*Hubei sobemo-like virus 15*
OR114813	CLCM-067	*Hubei sobemo-like virus 15*
OR114814	CLCM-072	*Hubei sobemo-like virus 15*
OR114815	CLCM-073	*Hubei sobemo-like virus 15*
OR114816	CLCM-012	*Hubei sobemo-like virus 15*
OR114817	CLCM-022	*Hubei sobemo-like virus 15*
OR114818	CLCM-031	*Hubei sobemo-like virus 15*
OR114819	CLCM-036	*Hubei sobemo-like virus 15*
OR114820	CLCM-037	*Hubei sobemo-like virus 15*
OR114821	CLCM-038	*Hubei sobemo-like virus 15*
OR114822	CLCM-053	*Hubei sobemo-like virus 15*
OR114823	CLCM-056	*Hubei sobemo-like virus 15*
OR114824	CLCM-057	*Hubei sobemo-like virus 15*
OR114825	CLCM-092	*Hubei sobemo-like virus 15*
OR114826	CLCM-096	*Hubei sobemo-like virus 15*
OR114827	CLCM-097	*Hubei sobemo-like virus 15*
OR114828	CLCM-098	*Hubei sobemo-like virus 15*
OR114829	CLCM-099	*Hubei sobemo-like virus 15*
OR114830	CLCM-106	*Hubei sobemo-like virus 15*
OR114831	CLCM-107	*Hubei sobemo-like virus 15*
OR114832	CLCM-111	*Hubei sobemo-like virus 15*
OR114833	CLCM-117	*Hubei sobemo-like virus 15*
OR114834	CLCM-118	*Hubei sobemo-like virus 15*
OR114835	CLCM-119	*Hubei sobemo-like virus 15*

OR114836	CLCM-120	*Hubei sobemo-like virus 15*
OR114837	CLCM-123	*Hubei sobemo-like virus 15*
OR114838	CLCM-127	*Hubei sobemo-like virus 15*
OR114839	CLCM-128	*Hubei sobemo-like virus 15*
OR114840	CLCM-130	*Hubei sobemo-like virus 15*
OR114841	CLCM-132	*Hubei sobemo-like virus 15*
OR114842	CLCM-134	*Hubei sobemo-like virus 15*
OR114843	CLCM-135	*Hubei sobemo-like virus 15*
OR114844	CLCM-136	*Hubei sobemo-like virus 15*
OR114845	CLCM-040	*Cheeloo tick virus 1*
OR114846	CLCM-089	*Hubei sobemo-like virus 15*
OR114847	CLCM-103	*Hubei sobemo-like virus 15*
OR114848	CLCM-040	*Cheeloo tick virus 2*
OR114849	CLCM-101	*Mivirus wuhanense*
OR114850	CLCM-060	*Cheeloo Jingmen-like virus*
OR114851	CLCM-069	*Cheeloo Jingmen-like virus*
OR114852	CLCM-071	*Cheeloo Jingmen-like virus*
OR114853	CLCM-077	*Cheeloo Jingmen-like virus*
OR114854	CLCM-079	*Cheeloo Jingmen-like virus*
OR114855	CLCM-080	*Cheeloo Jingmen-like virus*
OR114856	CLCM-011	*Cheeloo Jingmen-like virus*
OR114857	CLCM-013	*Cheeloo Jingmen-like virus*
OR114858	CLCM-014	*Cheeloo Jingmen-like virus*
OR114859	CLCM-016	*Cheeloo Jingmen-like virus*
OR114860	CLCM-018	*Cheeloo Jingmen-like virus*
OR114861	CLCM-019	*Cheeloo Jingmen-like virus*
OR114862	CLCM-022	*Cheeloo Jingmen-like virus*
OR114863	CLCM-026	*Cheeloo Jingmen-like virus*
OR114864	CLCM-028	*Cheeloo Jingmen-like virus*
OR114865	CLCM-029	*Cheeloo Jingmen-like virus*
OR114866	CLCM-002	*Cheeloo Jingmen-like virus*
OR114867	CLCM-030	*Cheeloo Jingmen-like virus*
OR114868	CLCM-031	*Cheeloo Jingmen-like virus*
OR114869	CLCM-034	*Cheeloo Jingmen-like virus*
OR114870	CLCM-035	*Cheeloo Jingmen-like virus*
OR114871	CLCM-037	*Cheeloo Jingmen-like virus*
OR114872	CLCM-040	*Cheeloo Jingmen-like virus*
OR114873	CLCM-041	*Cheeloo Jingmen-like virus*
OR114874	CLCM-042	*Cheeloo Jingmen-like virus*
OR114875	CLCM-043	*Cheeloo Jingmen-like virus*
OR114876	CLCM-004	*Cheeloo Jingmen-like virus*
OR114877	CLCM-045	*Cheeloo Jingmen-like virus*
OR114878	CLCM-046	*Cheeloo Jingmen-like virus*
OR114879	CLCM-047	*Cheeloo Jingmen-like virus*
OR114880	CLCM-050	*Cheeloo Jingmen-like virus*
OR114881	CLCM-052	*Cheeloo Jingmen-like virus*
OR114882	CLCM-055	*Cheeloo Jingmen-like virus*
OR114883	CLCM-056	*Cheeloo Jingmen-like virus*
OR114884	CLCM-057	*Cheeloo Jingmen-like virus*

OR114885	CLCM-058	*Cheeloo Jingmen-like virus*
OR114886	CLCM-006	*Cheeloo Jingmen-like virus*
OR114887	CLCM-059	*Cheeloo Jingmen-like virus*
OR114888	CLCM-007	*Cheeloo Jingmen-like virus*
OR114889	CLCM-008	*Cheeloo Jingmen-like virus*
OR114890	CLCM-083	*Cheeloo Jingmen-like virus*
OR114891	CLCM-086	*Cheeloo Jingmen-like virus*
OR114892	CLCM-088	*Cheeloo Jingmen-like virus*
OR114893	CLCM-089	*Cheeloo Jingmen-like virus*
OR114894	CLCM-090	*Cheeloo Jingmen-like virus*
OR114895	CLCM-091	*Cheeloo Jingmen-like virus*
OR114896	CLCM-094	*Cheeloo Jingmen-like virus*
OR114897	CLCM-117	*Cheeloo Jingmen-like virus*
OR114898	CLCM-119	*Cheeloo Jingmen-like virus*
OR114899	CLCM-004	*Mogiana tick virus*
OR114900	CLCM-004	*Mogiana tick virus*
OR114901	CLCM-015	*Cheeloo Jingmen-like virus*
OR114902	CLCM-020	*Cheeloo Jingmen-like virus*
OR114903	CLCM-027	*Cheeloo Jingmen-like virus*
OR114904	CLCM-038	*Cheeloo Jingmen-like virus*
OR114905	CLCM-039	*Cheeloo Jingmen-like virus*
OR114906	CLCM-048	*Cheeloo Jingmen-like virus*
OR114907	CLCM-049	*Cheeloo Jingmen-like virus*
OR114908	CLCM-005	*Cheeloo Jingmen-like virus*
OR114909	CLCM-053	*Cheeloo Jingmen-like virus*
OR114910	CLCM-054	*Cheeloo Jingmen-like virus*
OR114911	CLCM-084	*Cheeloo Jingmen-like virus*
OR114912	CLCM-004	*Mogiana tick virus*
OR114913	CLCM-004	*Mogiana tick virus*
OR114914	CLCM-076	*Haemaphysalis flava iflavirus*
OR114915	CLCM-081	*Hubei tick virus 1*
OR114916	CLCM-075	*Qingdao tick iflavirus*
OR114917	CLCM-135	*Cheeloo ifla-like virus*
OR114918	CLCM-040	*Cheeloo orthomyxo-like virus*
OR114919	CLCM-067	*Thogotovirus thogotoense*
OR114920	CLCM-035	*Thogotovirus thogotoense*
OR114921	CLCM-035	*Thogotovirus thogotoense*
OR114922	CLCM-035	*Thogotovirus thogotoense*
OR114923	CLCM-035	*Thogotovirus thogotoense*
OR114924	CLCM-107	*Thogotovirus thogotoense*
OR114925	CLCM-040	*Cheeloo orthomyxo-like virus*
OR114926	CLCM-067	*Thogotovirus thogotoense*
OR114927	CLCM-067	*Thogotovirus thogotoense*
OR114928	CLCM-067	*Thogotovirus thogotoense*
OR114929	CLCM-067	*Thogotovirus thogotoense*
OR114930	CLCM-067	*Thogotovirus thogotoense*
OR114931	CLCM-035	*Thogotovirus thogotoense*
OR114932	CLCM-035	*Thogotovirus thogotoense*
OR114933	CLCM-107	*Thogotovirus thogotoense*

OR114934	CLCM-107	*Thogotovirus thogotoense*
OR114935	CLCM-107	*Thogotovirus thogotoense*
OR114936	CLCM-107	*Thogotovirus thogotoense*
OR114937	CLCM-107	*Thogotovirus thogotoense*
OR114938	CLCM-060	*Henan tick virus*
OR114939	CLCM-060	*Henan tick virus*
OR114940	CLCM-060	*Henan tick virus*
OR114941	CLCM-072	*Henan tick virus*
OR114942	CLCM-072	*Henan tick virus*
OR114943	CLCM-072	*Henan tick virus*
OR114944	CLCM-052	*Henan tick virus*
OR114945	CLCM-052	*Henan tick virus*
OR114946	CLCM-052	*Henan tick virus*
OR114947	CLCM-058	*Henan tick virus*
OR114948	CLCM-058	*Henan tick virus*
OR114949	CLCM-058	*Henan tick virus*
OR114950	CLCM-082	*Henan tick virus*
OR114951	CLCM-082	*Henan tick virus*
OR114952	CLCM-082	*Henan tick virus*
OR114953	CLCM-090	*Henan tick virus*
OR114954	CLCM-090	*Henan tick virus*
OR114955	CLCM-090	*Henan tick virus*
OR114956	CLCM-096	*Henan tick virus*
OR114957	CLCM-096	*Henan tick virus*
OR114958	CLCM-096	*Henan tick virus*
OR114959	CLCM-097	*Henan tick virus*
OR114960	CLCM-097	*Henan tick virus*
OR114961	CLCM-097	*Henan tick virus*
OR114962	CLCM-099	*Henan tick virus*
OR114963	CLCM-099	*Henan tick virus*
OR114964	CLCM-099	*Henan tick virus*
OR114965	CLCM-130	*Henan tick virus*
OR114966	CLCM-130	*Henan tick virus*
OR114967	CLCM-130	*Henan tick virus*
OR114968	CLCM-132	*Henan tick virus*
OR114969	CLCM-132	*Henan tick virus*
OR114970	CLCM-132	*Henan tick virus*
OR114971	CLCM-083	*Henan tick virus*
OR114972	CLCM-083	*Henan tick virus*
OR114973	CLCM-065	*Shanxi tick virus 2*
OR114974	CLCM-065	*Shanxi tick virus 2*
OR114975	CLCM-065	*Shanxi tick virus 2*
OR114976	CLCM-037	*Shanxi tick virus 2*
OR114977	CLCM-037	*Shanxi tick virus 2*
OR114978	CLCM-037	*Shanxi tick virus 2*
OR114979	CLCM-100	*Shanxi tick virus 2*
OR114980	CLCM-100	*Shanxi tick virus 2*
OR114981	CLCM-100	*Shanxi tick virus 2*
OR114982	CLCM-119	*Shanxi tick virus 2*

OR114983	CLCM-119	*Shanxi tick virus 2*
OR114984	CLCM-119	*Shanxi tick virus 2*
OR114985	CLCM-083	*Henan tick virus*
OR114986	CLCM-068	*Uukuvirus dabieshanense*
OR114987	CLCM-068	*Uukuvirus dabieshanense*
OR114988	CLCM-070	*Uukuvirus dabieshanense*
OR114989	CLCM-070	*Uukuvirus dabieshanense*
OR114990	CLCM-071	*Uukuvirus dabieshanense*
OR114991	CLCM-071	*Uukuvirus dabieshanense*
OR114992	CLCM-078	*Uukuvirus dabieshanense*
OR114993	CLCM-078	*Uukuvirus dabieshanense*
OR114994	CLCM-081	*Uukuvirus dabieshanense*
OR114995	CLCM-081	*Uukuvirus dabieshanense*
OR114996	CLCM-081	*Changping Tick Virus 1*
OR114997	CLCM-011	*Uukuvirus dabieshanense*
OR114998	CLCM-011	*Uukuvirus dabieshanense*
OR114999	CLCM-017	*Uukuvirus dabieshanense*
OR115000	CLCM-017	*Uukuvirus dabieshanense*
OR115001	CLCM-019	*Uukuvirus dabieshanense*
OR115002	CLCM-019	*Uukuvirus dabieshanense*
OR115003	CLCM-021	*Uukuvirus dabieshanense*
OR115004	CLCM-021	*Uukuvirus dabieshanense*
OR115005	CLCM-030	*Uukuvirus dabieshanense*
OR115006	CLCM-030	*Uukuvirus dabieshanense*
OR115007	CLCM-081	*Changping Tick Virus 1*
OR115008	CLCM-031	*Uukuvirus dabieshanense*
OR115009	CLCM-031	*Uukuvirus dabieshanense*
OR115010	CLCM-032	*Uukuvirus dabieshanense*
OR115011	CLCM-032	*Uukuvirus dabieshanense*
OR115012	CLCM-033	*Uukuvirus dabieshanense*
OR115013	CLCM-033	*Uukuvirus dabieshanense*
OR115014	CLCM-003	*Uukuvirus dabieshanense*
OR115015	CLCM-003	*Uukuvirus dabieshanense*
OR115016	CLCM-034	*Uukuvirus dabieshanense*
OR115017	CLCM-034	*Uukuvirus dabieshanense*
OR115018	CLCM-037	*Uukuvirus dabieshanense*
OR115019	CLCM-037	*Uukuvirus dabieshanense*
OR115020	CLCM-048	*Uukuvirus dabieshanense*
OR115021	CLCM-048	*Uukuvirus dabieshanense*
OR115022	CLCM-054	*Uukuvirus dabieshanense*
OR115023	CLCM-054	*Uukuvirus dabieshanense*
OR115024	CLCM-055	*Uukuvirus dabieshanense*
OR115025	CLCM-055	*Uukuvirus dabieshanense*
OR115026	CLCM-056	*Uukuvirus dabieshanense*
OR115027	CLCM-056	*Uukuvirus dabieshanense*
OR115028	CLCM-057	*Uukuvirus dabieshanense*
OR115029	CLCM-057	*Uukuvirus dabieshanense*
OR115030	CLCM-058	*Uukuvirus dabieshanense*
OR115031	CLCM-058	*Uukuvirus dabieshanense*

OR115032	CLCM-059	*Uukuvirus dabieshanense*
OR115033	CLCM-059	*Uukuvirus dabieshanense*
OR115034	CLCM-082	*Uukuvirus dabieshanense*
OR115035	CLCM-082	*Uukuvirus dabieshanense*
OR115036	CLCM-083	*Uukuvirus dabieshanense*
OR115037	CLCM-083	*Uukuvirus dabieshanense*
OR115038	CLCM-084	*Uukuvirus dabieshanense*
OR115039	CLCM-084	*Uukuvirus dabieshanense*
OR115040	CLCM-086	*Uukuvirus dabieshanense*
OR115041	CLCM-086	*Uukuvirus dabieshanense*
OR115042	CLCM-087	*Uukuvirus dabieshanense*
OR115043	CLCM-087	*Uukuvirus dabieshanense*
OR115044	CLCM-089	*Uukuvirus dabieshanense*
OR115045	CLCM-089	*Uukuvirus dabieshanense*
OR115046	CLCM-091	*Uukuvirus dabieshanense*
OR115047	CLCM-091	*Uukuvirus dabieshanense*
OR115048	CLCM-092	*Uukuvirus dabieshanense*
OR115049	CLCM-092	*Uukuvirus dabieshanense*
OR115050	CLCM-093	*Uukuvirus dabieshanense*
OR115051	CLCM-093	*Uukuvirus dabieshanense*
OR115052	CLCM-094	*Uukuvirus dabieshanense*
OR115053	CLCM-094	*Uukuvirus dabieshanense*
OR115054	CLCM-095	*Uukuvirus dabieshanense*
OR115055	CLCM-095	*Uukuvirus dabieshanense*
OR115056	CLCM-096	*Uukuvirus dabieshanense*
OR115057	CLCM-096	*Uukuvirus dabieshanense*
OR115058	CLCM-097	*Uukuvirus dabieshanense*
OR115059	CLCM-097	*Uukuvirus dabieshanense*
OR115060	CLCM-098	*Uukuvirus dabieshanense*
OR115061	CLCM-098	*Uukuvirus dabieshanense*
OR115062	CLCM-099	*Uukuvirus dabieshanense*
OR115063	CLCM-099	*Uukuvirus dabieshanense*
OR115064	CLCM-100	*Uukuvirus dabieshanense*
OR115065	CLCM-100	*Uukuvirus dabieshanense*
OR115066	CLCM-101	*Uukuvirus dabieshanense*
OR115067	CLCM-101	*Uukuvirus dabieshanense*
OR115068	CLCM-103	*Uukuvirus dabieshanense*
OR115069	CLCM-103	*Uukuvirus dabieshanense*
OR115070	CLCM-104	*Uukuvirus dabieshanense*
OR115071	CLCM-104	*Uukuvirus dabieshanense*
OR115072	CLCM-105	*Uukuvirus dabieshanense*
OR115073	CLCM-105	*Uukuvirus dabieshanense*
OR115074	CLCM-106	*Uukuvirus dabieshanense*
OR115075	CLCM-106	*Uukuvirus dabieshanense*
OR115076	CLCM-107	*Uukuvirus dabieshanense*
OR115077	CLCM-107	*Uukuvirus dabieshanense*
OR115078	CLCM-108	*Uukuvirus dabieshanense*
OR115079	CLCM-108	*Uukuvirus dabieshanense*
OR115080	CLCM-109	*Uukuvirus dabieshanense*

OR115081	CLCM-109	*Uukuvirus dabieshanense*
OR115082	CLCM-111	*Uukuvirus dabieshanense*
OR115083	CLCM-111	*Uukuvirus dabieshanense*
OR115084	CLCM-114	*Uukuvirus dabieshanense*
OR115085	CLCM-114	*Uukuvirus dabieshanense*
OR115086	CLCM-116	*Uukuvirus dabieshanense*
OR115087	CLCM-116	*Uukuvirus dabieshanense*
OR115088	CLCM-117	*Uukuvirus dabieshanense*
OR115089	CLCM-117	*Uukuvirus dabieshanense*
OR115090	CLCM-121	*Uukuvirus dabieshanense*
OR115091	CLCM-121	*Uukuvirus dabieshanense*
OR115092	CLCM-122	*Uukuvirus dabieshanense*
OR115093	CLCM-122	*Uukuvirus dabieshanense*
OR115094	CLCM-123	*Uukuvirus dabieshanense*
OR115095	CLCM-123	*Uukuvirus dabieshanense*
OR115096	CLCM-124	*Uukuvirus dabieshanense*
OR115097	CLCM-124	*Uukuvirus dabieshanense*
OR115098	CLCM-125	*Uukuvirus dabieshanense*
OR115099	CLCM-125	*Uukuvirus dabieshanense*
OR115100	CLCM-126	*Uukuvirus dabieshanense*
OR115101	CLCM-126	*Uukuvirus dabieshanense*
OR115102	CLCM-127	*Uukuvirus dabieshanense*
OR115103	CLCM-127	*Uukuvirus dabieshanense*
OR115104	CLCM-128	*Uukuvirus dabieshanense*
OR115105	CLCM-128	*Uukuvirus dabieshanense*
OR115106	CLCM-129	*Uukuvirus dabieshanense*
OR115107	CLCM-129	*Uukuvirus dabieshanense*
OR115108	CLCM-130	*Uukuvirus dabieshanense*
OR115109	CLCM-130	*Uukuvirus dabieshanense*
OR115110	CLCM-131	*Uukuvirus dabieshanense*
OR115111	CLCM-131	*Uukuvirus dabieshanense*
OR115112	CLCM-132	*Uukuvirus dabieshanense*
OR115113	CLCM-132	*Uukuvirus dabieshanense*
OR115114	CLCM-133	*Uukuvirus dabieshanense*
OR115115	CLCM-133	*Uukuvirus dabieshanense*
OR115116	CLCM-134	*Uukuvirus dabieshanense*
OR115117	CLCM-134	*Uukuvirus dabieshanense*
OR115118	CLCM-135	*Uukuvirus dabieshanense*
OR115119	CLCM-135	*Uukuvirus dabieshanense*
OR115120	CLCM-136	*Uukuvirus dabieshanense*
OR115121	CLCM-136	*Uukuvirus dabieshanense*
OR115122	CLCM-110	*Uukuvirus dabieshanense*
OR115123	CLCM-100	*Lihan uukuvirus*
OR115124	CLCM-075	*Okutama tick virus*
OR115125	CLCM-075	*Okutama tick virus*
OR115126	CLCM-079	*Cheeloo phenui-like virus*
OR115127	CLCM-013	*Cheeloo phenui-like virus*
OR115128	CLCM-018	*Cheeloo phenui-like virus*
OR115129	CLCM-035	*Cheeloo phenui-like virus*

OR115130	CLCM-058	*Cheeloo phenui-like virus*
OR115131	CLCM-059	*Cheeloo phenui-like virus*
OR115132	CLCM-078	*Tick phlebovirus*
OR115133	CLCM-078	*Tick phlebovirus*
OR115134	CLCM-053	*Cheeloo uukuvirus*
OR115135	CLCM-053	*Cheeloo uukuvirus*
OR115136	CLCM-095	*Brown dog tick phlebovirus 1*
OR115137	CLCM-095	*Brown dog tick phlebovirus 1*
OR115138	CLCM-084	*Bandavirus dabieense*
OR115139	CLCM-084	*Bandavirus dabieense*
OR115140	CLCM-084	*Bandavirus dabieense*
OR115141	CLCM-096	*Bandavirus dabieense*
OR115142	CLCM-096	*Bandavirus dabieense*
OR115143	CLCM-096	*Bandavirus dabieense*
OR115144	CLCM-097	*Bandavirus dabieense*
OR115145	CLCM-097	*Bandavirus dabieense*
OR115146	CLCM-097	*Bandavirus dabieense*
OR115147	CLCM-099	*Bandavirus dabieense*
OR115148	CLCM-063	*Uukuvirus dabieshanense*
OR115149	CLCM-063	*Uukuvirus dabieshanense*
OR115150	CLCM-064	*Uukuvirus dabieshanense*
OR115151	CLCM-064	*Uukuvirus dabieshanense*
OR115152	CLCM-066	*Uukuvirus dabieshanense*
OR115153	CLCM-066	*Uukuvirus dabieshanense*
OR115154	CLCM-110	*Uukuvirus dabieshanense*
OR115155	CLCM-100	*Lihan uukuvirus*
OR115156	CLCM-099	*Bandavirus dabieense*
OR115157	CLCM-099	*Bandavirus dabieense*
OR148387	CLCM-061	*Cheeloo toti-like virus 1*
OR148388	CLCM-049	*Cheeloo toti-like virus 2*
OR148389	CLCM-053	*Cheeloo toti-like virus 2*
OR148390	CLCM-062	*Cheeloo luteovirus 2*
OR148391	CLCM-062	*Cheeloo tick virus 3*
OR148392	CLCM-071	*Cheeloo tick virus 3*
OR148393	CLCM-072	*Cheeloo tick virus 3*
OR148394	CLCM-075	*Cheeloo tick virus 3*
OR148395	CLCM-076	*Cheeloo luteovirus 3*
OR148396	CLCM-030	*Cheeloo tick virus 3*
OR148397	CLCM-039	*Cheeloo tick virus 3*
OR148398	CLCM-053	*Cheeloo tick virus 3*
OR148399	CLCM-056	*Cheeloo tick virus 3*
OR148400	CLCM-097	*Cheeloo luteovirus 1*
OR148401	CLCM-105	*Cheeloo luteovirus 1*
OR148402	CLCM-107	*Cheeloo tick virus 3*
OR148403	CLCM-115	*Cheeloo tick virus 3*
OR148404	CLCM-120	*Cheeloo tick virus 3*
OR148405	CLCM-131	*Cheeloo tick virus 3*
OR148406	CLCM-133	*Cheeloo tick virus 3*
OR148407	CLCM-040	*Caligrhavirus salmonlouse*

OR148408	CLCM-101	*Alpharicinrhavirus wuhan*
OR148409	CLCM-040	*Caligrhavirus lepeophtheirus*
OR148410	CLCM-018	*Xiangxi Parti tick virus 1*
OR148411	CLCM-134	*Acyrthosiphon pisum virus*
OR148412	CLCM-135	*Acyrthosiphon pisum virus*
OR148413	CLCM-136	*Acyrthosiphon pisum virus*
OR148414	CLCM-040	*Cheeloo partiti-like virus*
OR148415	CLCM-017	*Xiangxi Parti tick virus 1*
OR148416	CLCM-019	*Xiangxi Parti tick virus 1*
OR148417	CLCM-097	*Xiangxi Parti tick virus 1*
OR148418	CLCM-099	*Cheeloo luteovirus 1*
OR148419	CLCM-071	*Cheeloo tombus-like virus*
OR148420	CLCM-134	*Aphid lethal paralysis virus*
OR148421	CLCM-080	*Cheeloo triatovirus 2*
OR148422	CLCM-103	*Cheeloo triatovirus 1*
OR148423	CLCM-040	*Lepeophtheirus virus LS24*
OR148424	CLCM-040	*Cheeloo mononegavirus*
OR148425	CLCM-135	*Cheeloo permutotetra-like virus*
OR148426	CLCM-100	*Alpharicinrhavirus wuhan*
OR148428	CLCM-072	*Cheeloo virga-like virus*

Supplementary Table 3. The prevalence with 95% confidence interval (CI) of viruses in two clades

Class	Species	Positive numbers	Total numbers	Prevalence (%) (95% CI)
Overall	*Hepelivirales* sp.	68	136	50.00 (41.64–58.36)
Overall	*Cheeloo Jingmen-like virus*	60	136	44.12 (35.82–52.42)
Overall	*Hubei sobemo-like virus 15*	35	136	25.74 (18.43–33.05)
Overall	*Cheeloo noda-like virus 2*	30	136	22.06 (15.13–28.99)
Overall	*Cheeloo tick virus 3*	13	136	9.56 (4.64–14.48)
Overall	*Cheeloo noda-like virus 3*	12	136	8.82 (4.08–13.57)
Overall	*Henan tick virus*	12	136	8.82 (4.08–13.57)
Overall	*Cheelo phenui-like virus*	6	136	4.41 (0.98–7.85)
Overall	*Shanxi tick virus 2*	4	136	2.94 (0.12–5.77)
Overall	*Cheeloo toti-like virus 2*	2	136	1.47 (-0.54–3.48)
Clade 1	*Uukuvirus dabieshanense*	71	100	71 (62.15–79.85)
Clade 1	*Bandavirus dabieense*	4	100	4 (0.18–7.82)
Clade 1	*Xiangxi Parti tick virus 1*	4	100	4 (0.18–7.82)
Clade 1	*Acyrthosiphon pisum virus*	3	100	3 (-0.33–6.33)
Clade 1	*Cheeloo luteovirus 1*	3	100	3 (-0.33–6.33)
Clade 1	*Thogotovirus thogotoense*	3	100	3 (-0.33–6.33)
Clade 1	*Alpharicinrhavirus wuhan*	2	100	2 (-0.73–4.73)
Clade 1	*Aphid lethal paralysis virus*	1	100	1 (-0.94–2.94)
Clade 1	*Brown dog tick phlebovirus 1*	1	100	1 (-0.94–2.94)
Clade 1	*Changping Tick Virus 1*	1	100	1 (-0.94–2.94)
Clade 1	*Cheeloo ifla-like virus*	1	100	1 (-0.94–2.94)
Clade 1	*Cheeloo luteovirus 2*	1	100	1 (-0.94–2.94)
Clade 1	*Cheeloo permutotetra-like virus*	1	100	1 (-0.94–2.94)
Clade 1	*Cheeloo tombus-like virus*	1	100	1 (-0.94–2.94)
Clade 1	*Cheeloo triatovirus 1*	1	100	1 (-0.94–2.94)
Clade 1	*Cheeloo uukuvirus*	1	100	1 (-0.94–2.94)
Clade 1	*Hubei tick virus 1*	1	100	1 (-0.94–2.94)
Clade 1	*Lihan uukuvirus*	1	100	1 (-0.94–2.94)
Clade 1	*Mivirus wuhanense*	1	100	1 (-0.94–2.94)
Clade 1	*Tick phlebovirus*	1	100	1 (-0.94–2.94)
Clade 2	*Huangpi orthonairovirus*	3	36	8.33 (-0.65–17.32)
Clade 2	*Cheeloo noda-like virus 1*	2	36	5.56 (-1.89–13)
Clade 2	*Caligrhavirus lepeophtheirus*	1	36	2.78 (-2.56–8.12)
Clade 2	*Caligrhavirus salmonlouse*	1	36	2.78 (-2.56–8.12)
Clade 2	*Cheeloo luteovirus 3*	1	36	2.78 (-2.56–8.12)
Clade 2	*Cheeloo mononegavirus*	1	36	2.78 (-2.56–8.12)
Clade 2	*Cheeloo orthomyxo-like virus*	1	36	2.78 (-2.56–8.12)
Clade 2	*Cheeloo partiti-like virus*	1	36	2.78 (-2.56–8.12)
Clade 2	*Cheeloo tick virus 1*	1	36	2.78 (-2.56–8.12)
Clade 2	*Cheeloo tick virus 2*	1	36	2.78 (-2.56–8.12)
Clade 2	*Cheeloo toti-like virus 1*	1	36	2.78 (-2.56–8.12)
Clade 2	*Cheeloo triatovirus 2*	1	36	2.78 (-2.56–8.12)

Clade 2	*Cheeloo virga-like virus*	1	36	2.78 (-2.56–8.12)
Clade 2	*Haemaphysalis flava iflavirus*	1	36	2.78 (-2.56–8.12)
Clade 2	*Lepeophtheirus virus LS24*	1	36	2.78 (-2.56–8.12)
Clade 2	*Mogiana tick virus*	1	36	2.78 (-2.56–8.12)
Clade 2	*Okutama tick virus*	1	36	2.78 (-2.56–8.12)
Clade 2	*Qingdao tick iflavirus*	1	36	2.78 (-2.56–8.12)

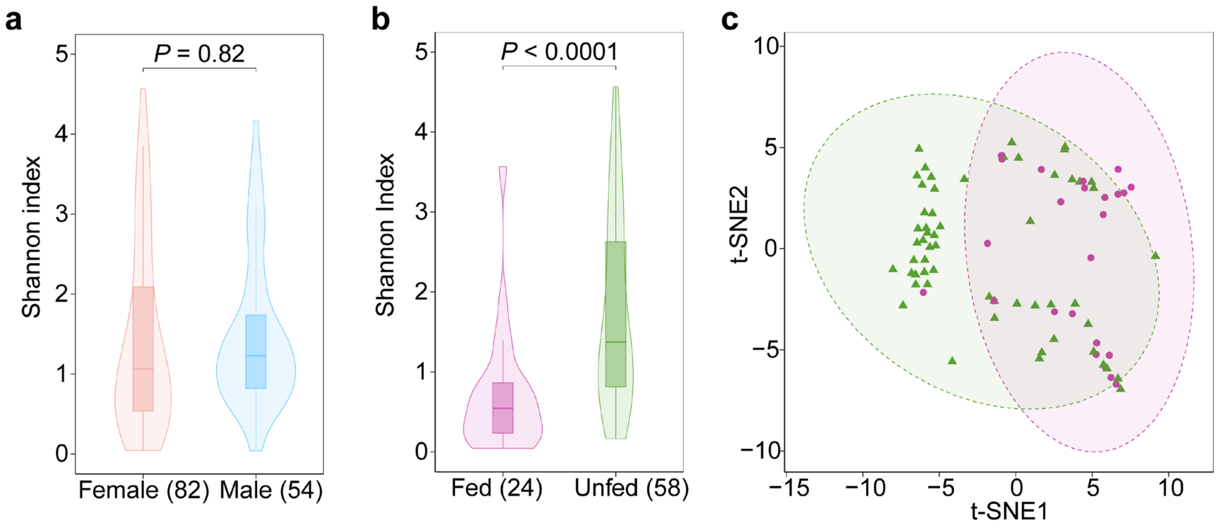

Supplementary Figure 1. The virome diversity. a, Shannon indexes of tick viromes were compared between female and male (n_{female}= 82, n_{male}= 54). **b**, Shannon indexes of tick viromes were compared between fed and unfed female ticks (n_{fed}= 24, n_{unfed}= 58). **c**, Between-group clustering of viromes between fed and unfed ticks of *H. longicornis* by t-SNE analysis. Boxplot elements: centre line, median; box limits, upper and lower quartiles; whiskers (error bars), the highest and lowest points within 1.5 interquartile range of the upper and lower quartiles. The *P*-value was calculated using a two-sided Wilcoxon rank-sum test.

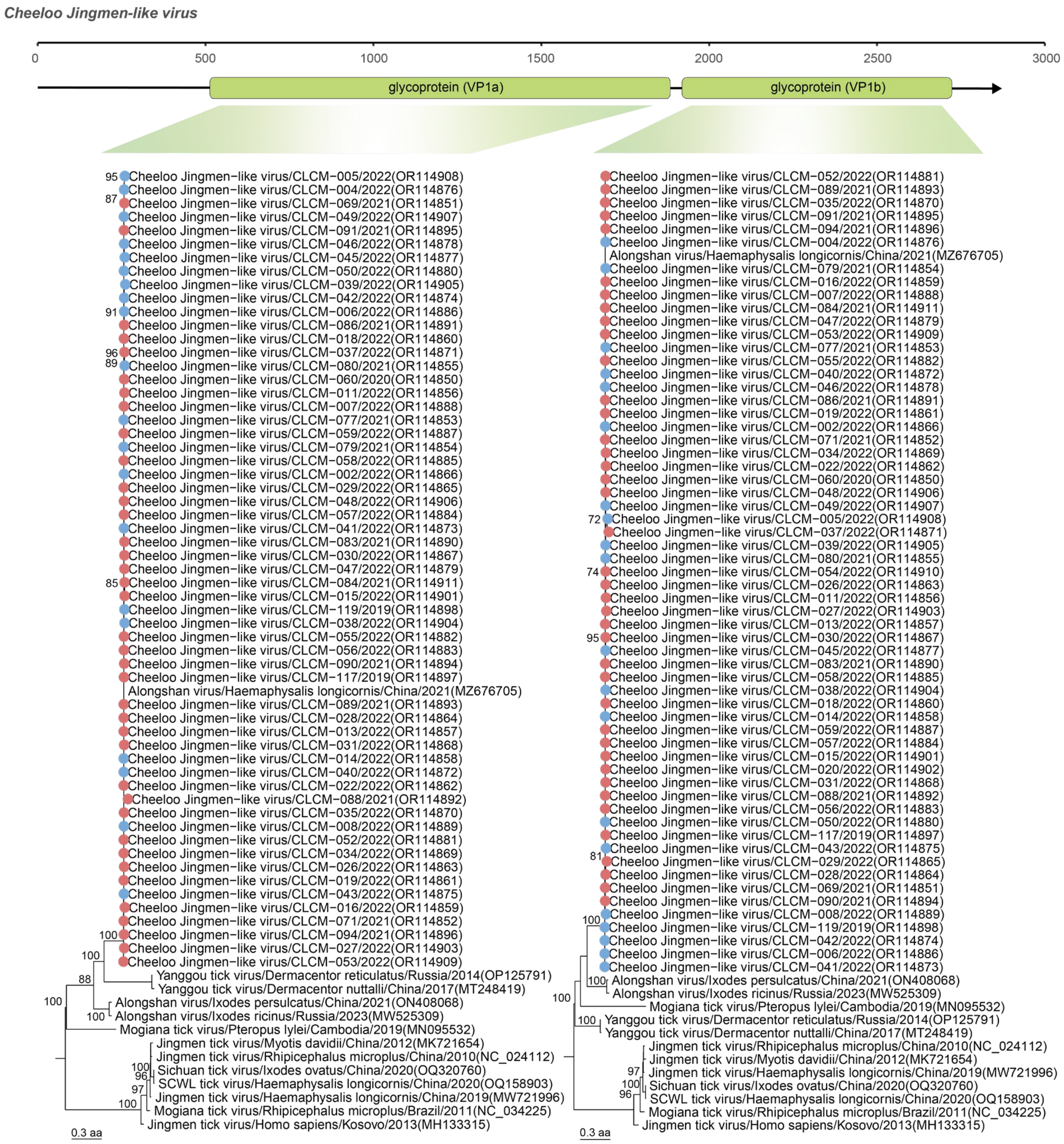

Supplementary Figure 2. Phylogenetic analysis of *Cheeloo Jingmen-like virus* based on glycoprotein. Genome organization of glycoprotein in *Cheeloo Jingmen-like virus* on the top. Phylogeny of viruses in the group of Jingmenvirus based on amino acid sequence of VP1a protein on the left side and VP1b protein on the right side.

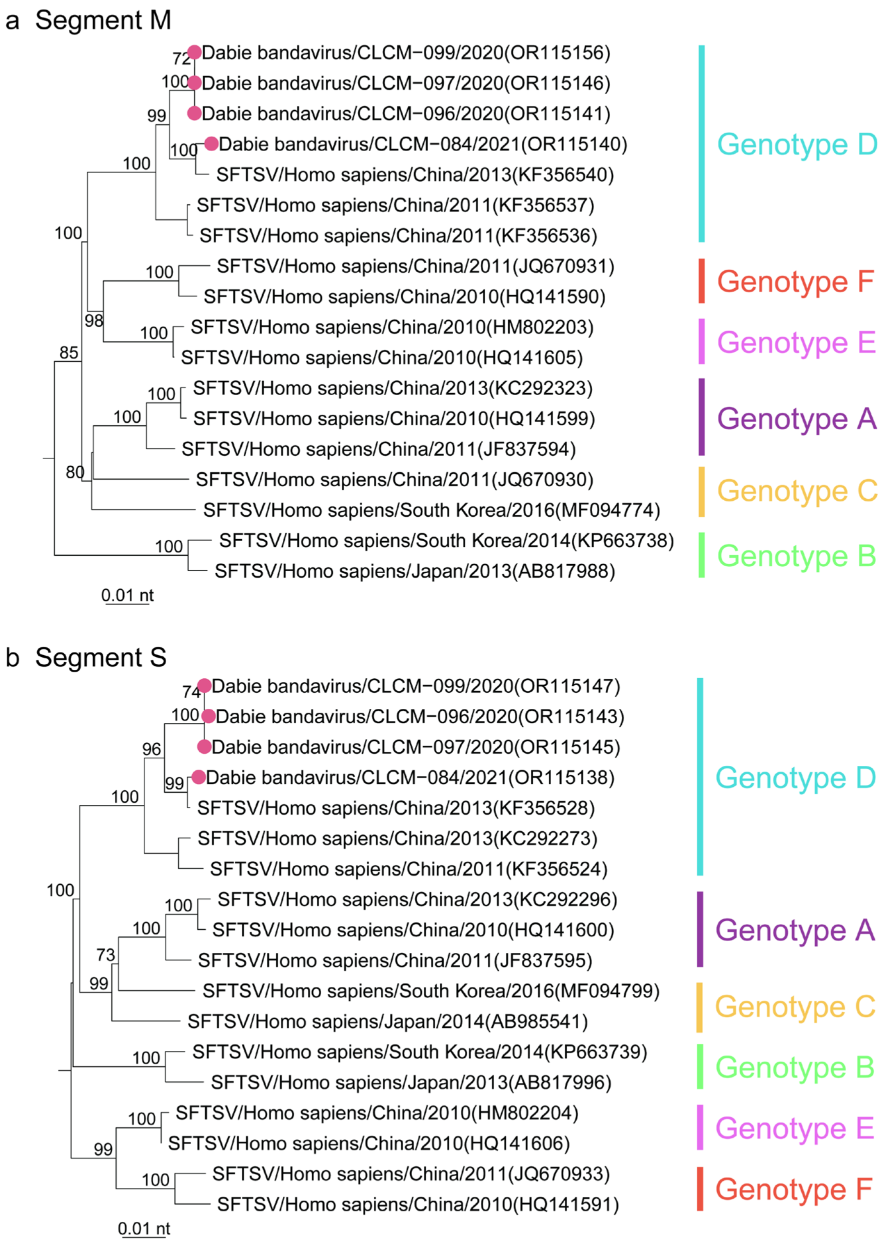

Supplementary Figure 3. Phylogenetic analysis of *Bandavirus dabieense*. Phylogeny of viruses of *Bandavirus dabieense* based on nucleotide sequences of M segment (**a**) and S segment (**b**).

a

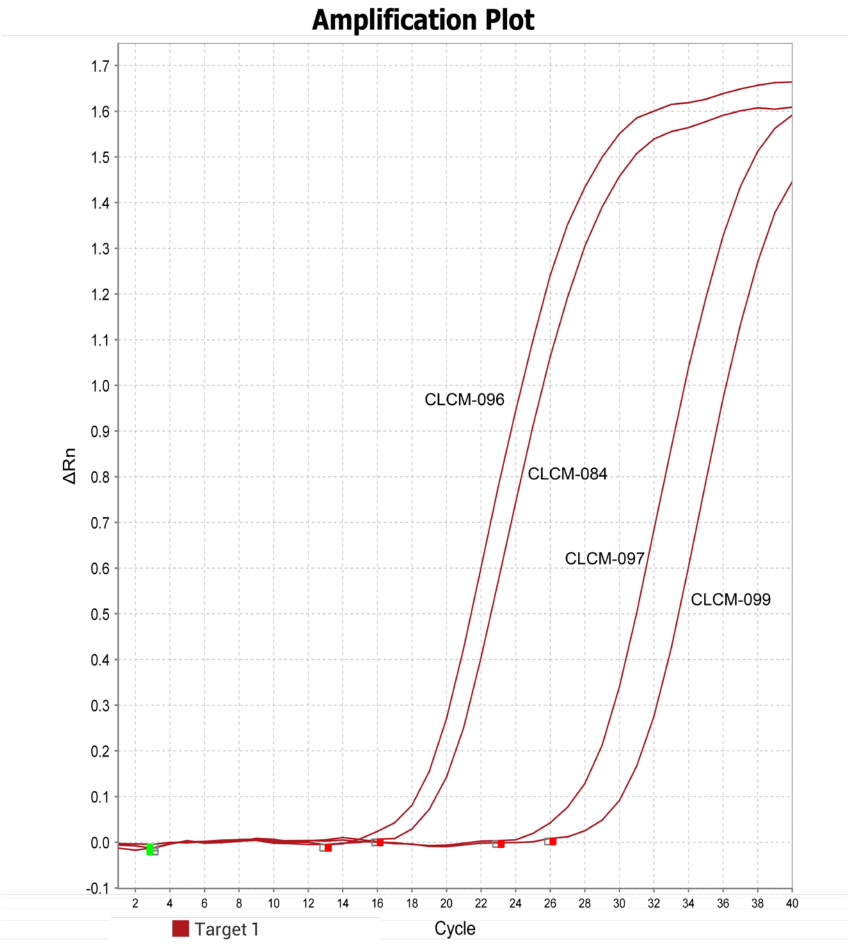

b

Primer	Sequence
SFTSV-F	AGCCTAATTGGATATGTCAAATTGC
SFTSV-R	CGGGTGAAGTGGCTGAAGG
SFTSV-P	AGCAGCAGCAGCAACCTCAGCAGC

Supplementary Figure 4. Validation of *Bandavirus dabieense*. The amplification plot of the tick samples positive for *Bandavirus dabieense* by real time RT-PCR test with cycle threshold (Ct) < 35 (a). The primers used for the real time RT-PCR test are listed in Table (b).

Uukuvirus dabieshanense

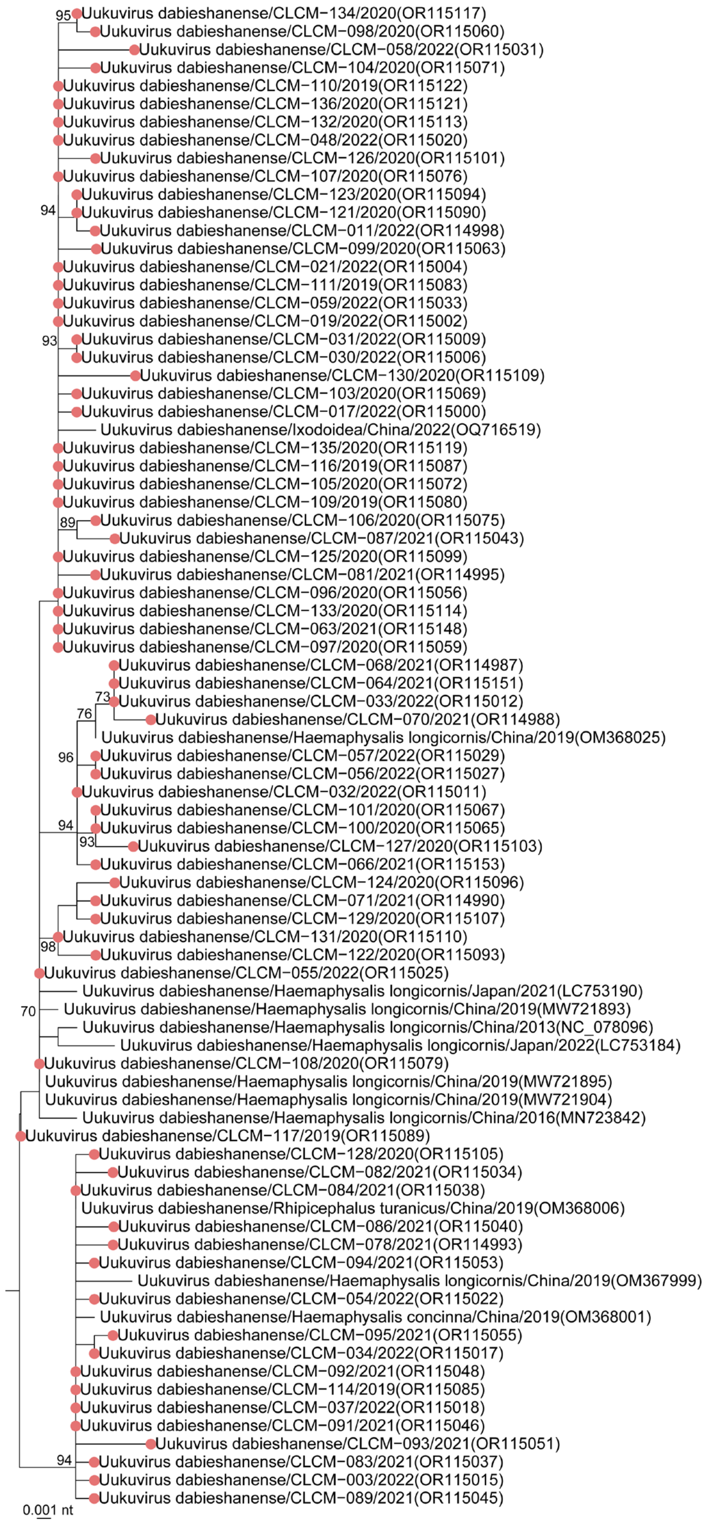

Supplementary Figure 5. Phylogeny of *Uukuvirus dabieshanense* based on nucleotide sequence of S gene.

Viruses in this study are marked by red solid circles.

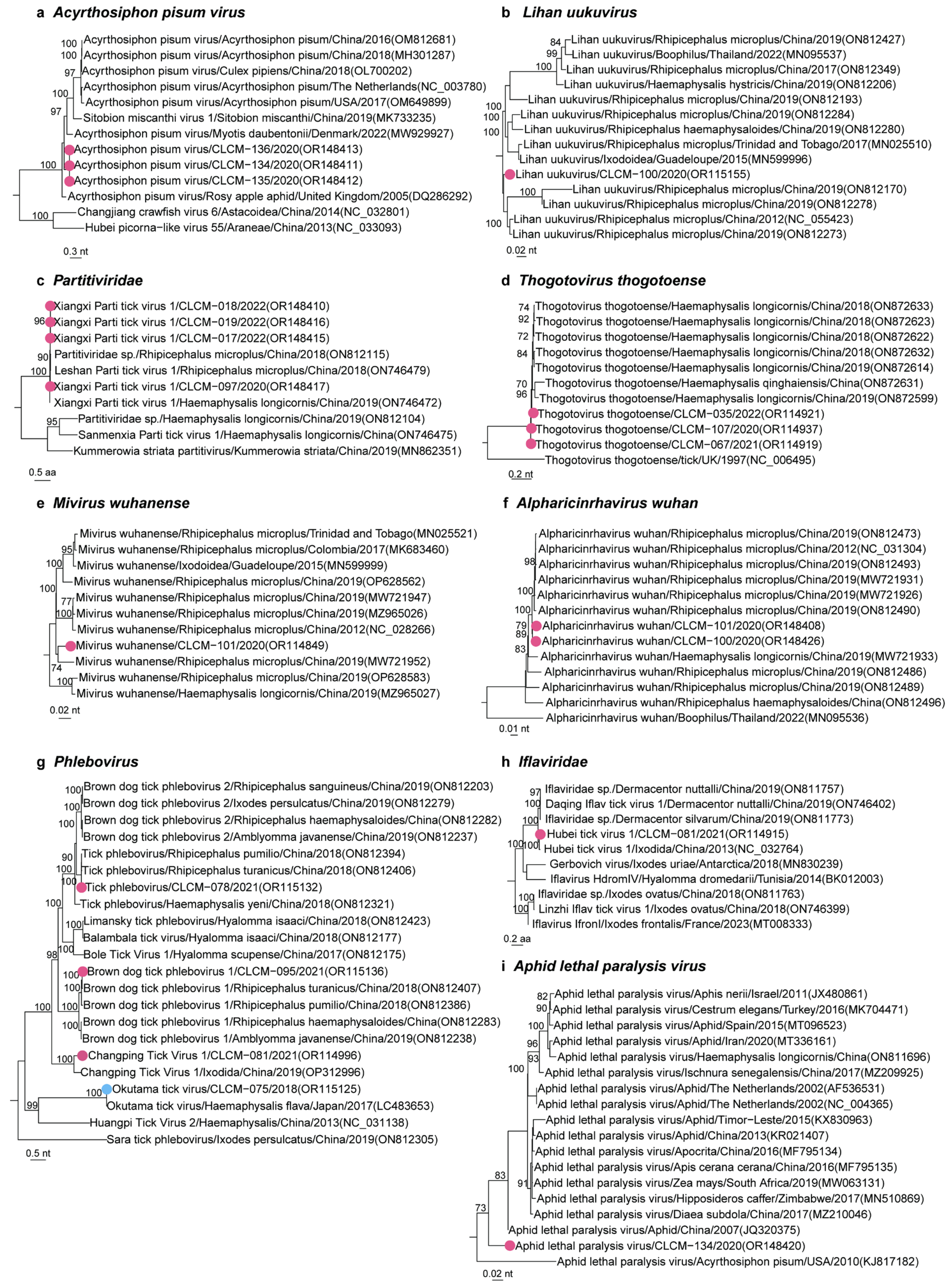

Supplementary Figure 6. Phylogenic analysis of known viruses in clade 1. a, Phylogeny of *Acyrthosiphon pisum virus* based on nucleotide sequence of RdRp gene. **b**, Phylogeny of *Lihan uukuvirus* based on nucleotide sequence of RdRp gene. **c**, Phylogeny of viruses in the family of *Partitiviridae* based on amino acid sequence of RdRp protein. **d**, Phylogeny of *Thogotovirus thogotoense* based on nucleotide sequence of PB1 gene. **e**, Phylogeny

of *Mivirus wuhanense* based on nucleotide sequence of RdRp gene. **f**, Phylogeny of *Alpharicinrhavirus wuhan* based on nucleotide sequence of RdRp gene. **g**, Phylogeny of viruses in the genus *Phlebovirus* based on nucleotide sequence of RdRp gene. **h**, Phylogeny of *Iflaviridae* based on amino acid sequence of RdRp protein. **i**, Phylogeny of *Aphid lethal paralysis virus* based on nucleotide sequence of RdRp gene.

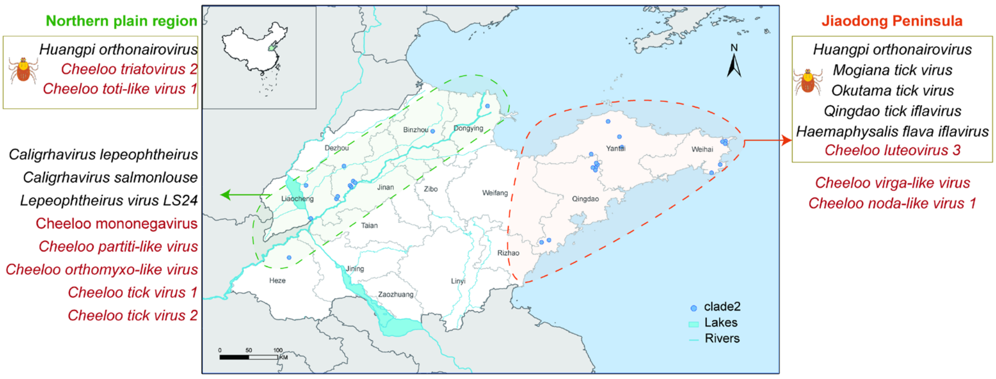

Supplementary Figure 7. The geographical distribution of *H. longicornis* in clade 2. The known viruses are indicated in black, while the newly-identified viruses in red.

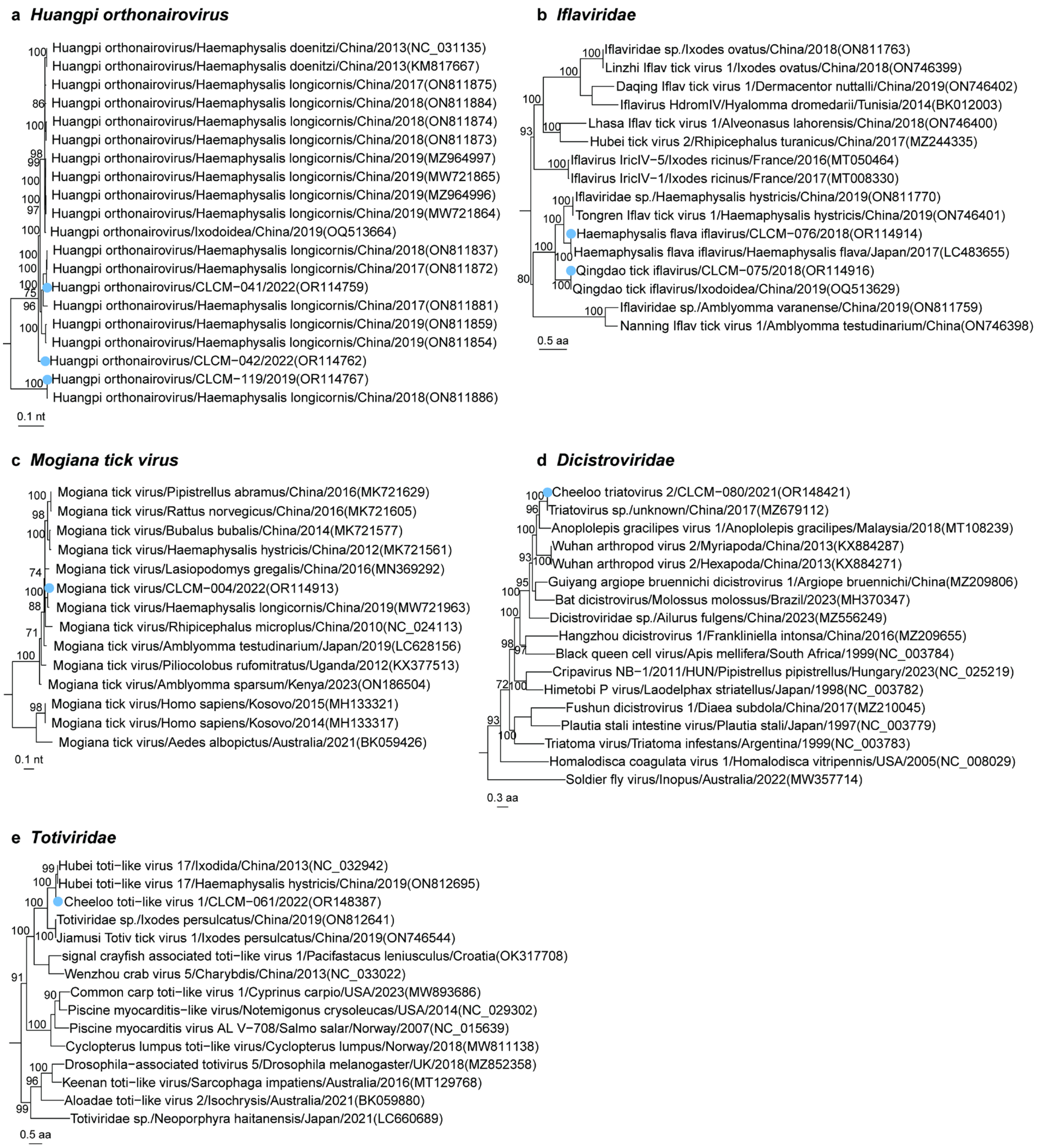

Supplementary Figure 8. Phylogenic analysis of arthropod-associated viruses in clade 2. **a**, Phylogeny of *Huangpi orthonairovirus* based on nucleotide sequence of RdRp gene. **b**, Phylogeny of viruses in the family *Iflaviridae* based on amino acid sequence of RdRp protein. **c**, Phylogeny of *Mogiana tick virus* based on nucleotide sequence of NS5 gene. **d**, Phylogeny of viruses in the family *Dicistroviridae* based on amino acid sequence of RdRp protein. **e**, Phylogeny of viruses in the family *Totiviridae* based on amino acid sequence of RdRp protein.
